# The impact of climate change and glacier mass loss on the hydrology in the Mont-Blanc massif

**DOI:** 10.1038/s41598-020-67379-7

**Published:** 2020-06-26

**Authors:** Léa Laurent, Jean-François Buoncristiani, Benjamin Pohl, Harry Zekollari, Daniel Farinotti, Matthias Huss, Jean-Louis Mugnier, Julien Pergaud

**Affiliations:** 1 BioGéosciences, UMR6282 CNRS/Université de Bourgogne Franche-Comté, Dijon, France; 2 Department of Geoscience and Remote Sensing, Delft University of Technology, Delft, The Netherlands; 3 Laboratoire de Glaciologie, Université Libre de Bruxelles, Brussels, Belgium; 4 Laboratory of Hydraulics, Hydrology and Glaciology (VAW), ETH Zürich, Zurich, Switzerland; 5 Swiss Federal Institute for Forest, Snow and Landscape Research (WSL), Birmensdorf, Switzerland; 6 Department of Geosciences, University of Fribourg, Fribourg, Switzerland; 7 Université Grenoble Alpes, Université Savoie Mont-Blanc, CNRS, ISTerre, Chambéry, France

**Keywords:** Cryospheric science, Projection and prediction, Hydrology, Hydrology, Environmental health

## Abstract

The Mont-Blanc massif, being iconic with its large glaciers and peaks of over 4,000 m, will experience a sharp increase in summer temperatures during the twenty-first century. By 2100, the impact of climate change on the cryosphere and hydrosphere in the Alps is expected to lead to a decrease in annual river discharge. In this work, we modelled the twenty-first century evolution of runoff in the Arve river, downstream of Mont-Blanc’s French side. For the first time for this region, we have forced a hydrological model with output from an ice-dynamical glacier model and 16 downscaled climate projections, under RCP4.5 and RCP8.5 scenarios. By 2100, under RCP8.5 (high-emission scenario), the winter discharge of the Arve river remains low but is expected to increase by 80% when compared to the beginning of the century. By contrast, the summer season, currently the most important discharge period, will be marked by a runoff decrease of approximately 40%. These changes are almost similar according to a scenario with a lower warming (RCP4.5) and are mostly driven by glacier retreat. These shifts will have significant downstream impacts on water quantity and quality, affecting hydroelectric generation, agriculture, forestry, tourism and aquatic ecosystems.

Global change and temperature increase are projected to lead to major environmental changes in mountainous regions^1^, including major changes in glacier extent^2,3^, permafrost^4^, ice and snow cover^5–7^, and vegetation^8,9^. In the Alps, the cryosphere is crucial for water storage and for contributing to the total discharge of the main major European rivers^10^. In this context, major changes are likely to occur in water discharge^11^ in and near these vulnerable regions, mostly decreasing runoff in summer and modifying water resources^12^. These changes could either be driven by (i) changes in precipitation^13,14^, especially in the proportion of liquid and solid water^15,16^, depending on the altitude of the 0 °C isotherm^17^; (ii) a general warming trend, increasing snow and ice melt, hereby contributing to glacier mass loss^18^, but also increasing evapotranspiration from the surface; (iii) glacier retreat in response to such warming, ultimately leading to changes in water discharge as the available ice reserves gradually decrease^11^. Of particular importance are the relative weight of each of these forcings over the coming years and decades, and their dependency on the greenhouse gas emission scenarios. These changes will have major effects on river discharges and water quality^19^, impacting hydropower generation, agriculture, forestry, tourism and aquatic ecosystems^8,20^.

Many studies have explored the impact of global warming and glacier retreat on glacier runoff, at global^21^ and regional scales (e.g., High Mountain Asia^22,23^, the Andes^24,25^ and the USA^26^). In the European Alps, glacier runoff evolution under climate change has already been investigated in Switzerland^27,28^ and Austria^29^ for example. In the French Alps, assessments of future changes are scarce, mostly focusing on a regional- to global scale (i.e. the entire European Alps)^30^. Hence, a spacial and temporal high resolution analysis of the impact of climate change and glacier retreat on discharge evolution is still lacking on the Mont-Blanc massif. In addition, studies projecting the long-term evolution runoff from glaciers in which ice dynamics are explicitly included do not exist for this region.

Projecting the hydrology in partially glaciated watershed requires modelled climate change projections corrected by downscaling techniques. These techniques are based on observational data, used as reference to correct some of the biases produced by numerical climate models and to adapt their coarse networks and partly biased results to the local and regional mountain context^31,32^. We focus here on the Mont-Blanc massif (Fig. 1b), an emblematic mountain range for mountaineers and tourists, and make use of the long and continuous periods of observational data that exist. The Massif is located on the border between France, Italy and Switzerland and shows several peaks above 4,000 m.a.s.l., of which Mont-Blanc is the highest peak in the Alps (4,810 m altitude). There, rapid environmental changes have already been recorded.

**Figure 1:**
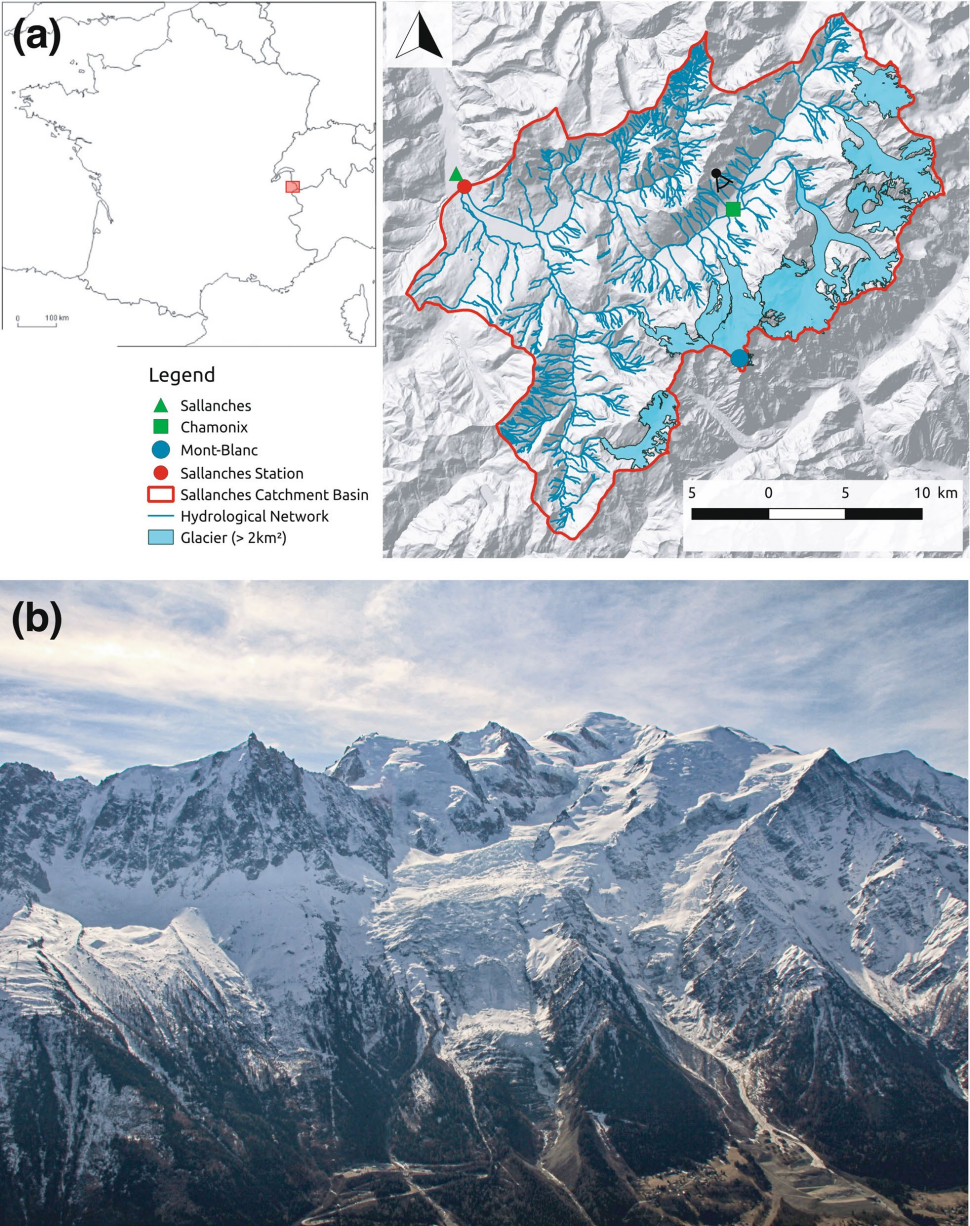
Study area. (**a**) Location of the Sallanches catchment in the French Alps and map of the studied catchment basin (QGis 2.18., https://www.qgis.org/fr/site/index.html). (**b**) The Mont-Blanc massif as seen from La Flégère cable car (black dot with view angle on the map).

In this study, we work on the Arve river watershed, where there are about 20 glaciers, eight of which now exceed two square kilometres (Fig. 1a). The studied catchment area is 570 km^2^, 16.0% is currently covered with ice against 16.6% in 1967^33^. In most years, catchment area is entirely snow covered in winter, whereas in summer only the upper parts of the ice-covered areas (i.e. mostly corresponding to the glacier’s accumulation zones) remains snow covered. Here, we produce and analyse the first high-resolution long-term simulations of water discharge for the Arve river at the Sallanches outlet, located downstream of the major glaciers on the French side of the Massif. We use a hydrological model^34^ forced by 16 downscaled and bias-corrected climate models^35^. These hydrological simulations explicitly consider the evolution of the glacier geometry throughout the century, as computed by a glaciological model (Supplementary Methods Sect. 1).

## A changing runoff in a changing climate

**Figure 2:**
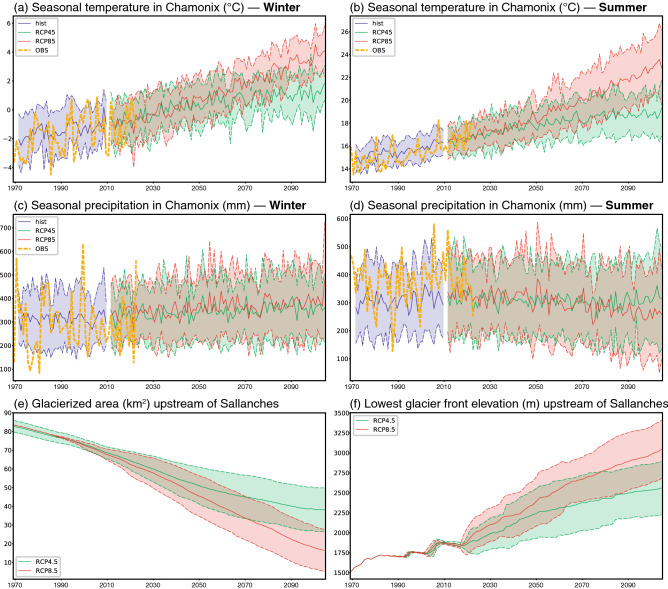
(**a**,**b**) Seasonal mean temperature in Chamonix in winter (DJF) and in summer (JJA) (°C). (**c**,**d**) Seasonal precipitation amounts in Chamonix in winter (DJF) and in summer (JJA) (mm). (**e**) Annual ice-covered area of the study area (km^2^). (**f**) Annual lowest glacier frontal elevation (m). Yellow curves: observations, period 1965–2018. Blue colours: historical (Hist) simulations, period 1965–2005. The solid curve shows the ensemble mean, the colour shading extends to ± 1 standard deviation to show model uncertainties. Green colours: the same for RCP4.5 simulations, period 2006–2100. Red colours: the same for RCP8.5 simulations, period 2006–2100.

The climate variables (temperature and precipitation) influencing water discharge in and around the Mont-Blanc Massif are firstly downscaled and post-corrected using the reference Chamonix weather station (1042 m.a.s.l., Fig. 2). These climate variables come from 16 general circulation models (GCMs) under representative concentration pathway (RCP) 4.5 and RCP8.5^36^. Catchment area, ice-covered proportion, slope and hypsometric curves are also computed to use as inputs to the hydrological model (Supplementary Methods Sect. 4). By construction, simulations from the historical runs fit observations at the Chamonix weather station in terms of statistical distribution. More specifically, they both depict a warming trend, at a comparable rate of roughly + 0.3 to + 0.4 °C per decade (i.e. + 1.5 °C between 1970 and 2010) in both winter (December–January–February) and summer (June–July–August). Future temperature evolution in winter is expected to lead to a warming of + 3.5 °C by 2100 against the pre-industrial era under RCP4.5 (i.e. + 2 °C during 1965–2018, which is our study period and constrained by observational data availability, and another + 1.5 °C between 2019 and 2100), and + 5.5 °C under RCP8.5 (Fig. 2a). These values are quite similar for the summer warming expected by the end of the century under RCP4.5, but a sensibly larger warming is anticipated for RCP8.5 (+ 8.5 °C against pre-industrial simulations, Fig. 2b). While RCP4.5 shows a slower increase in temperature for the last decades of the century, RCP8.5 produces an ever-accelerating warming trend. Although model uncertainties tend to be smaller in summer than in winter under historical radiative forcing, they increase over the current century, denoting differentiated climate sensitivity (that is, different warming under a similar radiative forcing). This is less true in winter, when uncertainties remain almost constant throughout the study period (Supplementary Fig. S9).

Temporal variations in seasonal precipitation are weaker compared to temperature, even though such apparent quasi-stability can conceal a significant decrease (or increase) in snow (or rain). In the Chamonix valley, this is especially true in winter, and in high-mountain environments in summer^17^. Over the next century, winter precipitation amounts show a weak positive trend (Fig. 2c), while the opposite occurs in summer (Fig. 2d). The magnitude of these changes remains nonetheless smaller than the inter-model spread, indicating that these results are less robust than the simulated changes in temperature.

As a result of these projected evolutions in temperature and precipitation, glaciers are expected to retreat dramatically by 2100^18^. Glacierized areas in the Arve basin are simulated to drop from roughly 90 km^2^ in the year 2004^33^ to 40 km^2^ and 20 km^2^ by the end of the century under RCP4.5 and RCP8.5, respectively (Fig. 2e and Supplementary Fig. S1). In the Mont-Blanc massif, this corresponds to a spectacular rise in glacier front elevations: from 1,500 m.a.s.l. under current conditions to 2,500 and 3,000 m.a.s.l. under RCP4.5 and RCP8.5 (average for the eight glaciers considered in this study, Fig. 2f).

**Figure 3:**
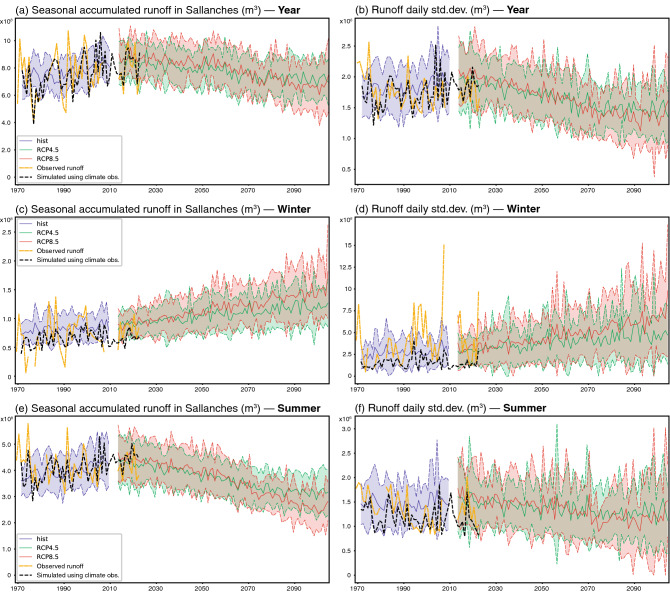
(**a**,**b**) Annual runoff and daily standard deviation of the runoff in Sallanches (m^3^). (**c**,**d**) Winter (DJF) runoff and daily standard deviation of the runoff in Sallanches (m^3^). (**e**,**f**) Summer (JJA) runoff and daily standard deviation of the runoff in Sallanches (m^3^). Yellow curves: observations, period 1965–2018. Black dotted curves: simulations using climate observations, period 1967–2018. Blue colours: historical (Hist) simulations, period 1967–2005. The solid curve shows the ensemble mean, the colour shading extends to ± 1 standard deviation to show model uncertainties. Green colours: the same for RCP4.5 simulations, period 2008–2100. Red colours: the same for RCP8.5 simulations, period 2008–2100.

Water discharge from the Arve river directly responds to this forcing (Fig. 3). As an input for hydrological model GSM-Socont, we only consider glaciers larger than 2 km^2^, as smaller glaciers represent only 15% of the total glacierized area and have therefore a small influence on the river runoff. Glaciated surfaces below 2 km^2^ are then considered as non-glaciated areas. GSM-Socont is a conceptual reservoir-based model producing hydrological discharge simulations at daily resolution (Supplementary Methods Sect. 4)^34^. Both seasonal means and day-to-day standard deviation are displayed, to characterize not only the overall evolution of the average discharge, but also the magnitude of its variability within each season. In spite of a very good skill (Supplementary Results Sect. 1), the hydrological model underestimates runoff variability from one season to another when forced by climate observations (Fig. 3d,f).

Runoff changes derived from climate simulations show contrasted seasonal changes between summer and winter (Fig. 3c,e). In winter, discharge is projected to increase substantially, hereby pursuing and enhancing the weak positive trend found in historical records (Fig. 3c). Under a high-emission scenario, winter discharge is expected to double from 1967 to 2100. This evolution is mostly driven by an increasing (decreasing) proportion of rainfall (snow), causing rapid runoff from the non-glacierized parts of the watershed, especially in its lowest parts. Day-to-day variability in winter is simulated to increase significantly (Fig. 3d), from 2.5 × 10^6^ m^3^ s^−1^ in 2005 to nearly 5 × 10^6^ m^3^ s^−1^ in 2100 under RCP4.5 and 7.5 × 10^6^ m^3^ s^−1^ under RCP8.5. The physical causes of these evolutions, which could be due to the zero-bounded distribution of the discharges, are discussed in the Supplementary Results (Sects. 3 and 4).

In summer, the seasonal mean runoff has significantly increased in the past decades (Fig. 3e), as a direct consequence of a general warming (Fig. 2b) that enhanced snow and ice melt, favouring glacier mass loss and retreat. Our simulations indicate that the maximum annual water release from glaciers (“peak water”) has been reached recently or should be reached in the coming 5–10 years (Fig. 3e). This is in line with the results of large-scale studies^21^. The coming decades are anticipated to be characterized by a marked summer runoff decrease of about a third under RCP8.5, denoting an increasing role of glacier retreat counteracting the effects of ongoing warming. Daily runoff variability in summer is likely to remain constant or become slightly lower throughout the century (Fig. 3f, Supplementary Results Sects. 3 and 4).

The annual runoff also peaks between 2010 and 2030 (i.e. “peak water”). After reaching a high of about 9 × 10^8^ m^3^ s^−1^ in 2008, runoff is expected to decrease to about 7 × 10^8^ m^3^ s^−1^ by 2100. This is mostly driven by changes in the summer runoff.

**Figure 4:**
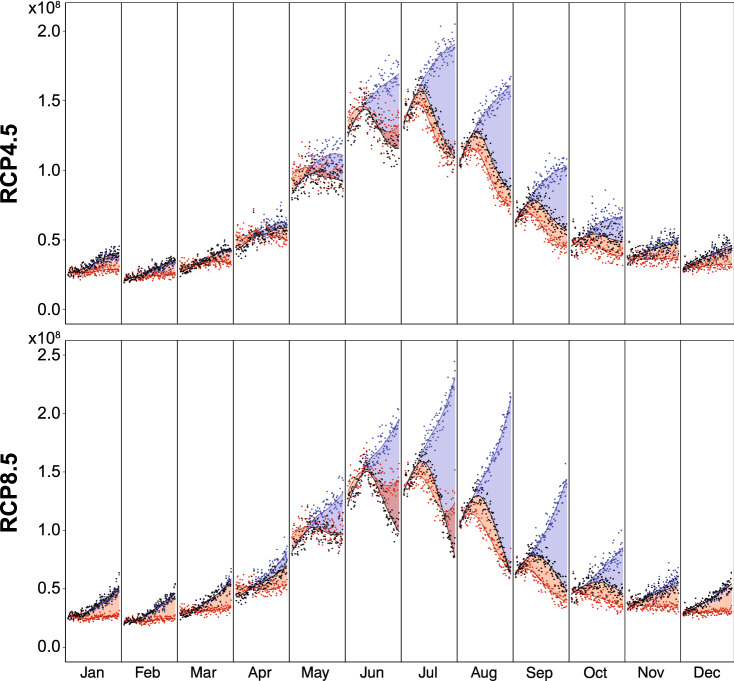
Monthly total runoff in Sallanches (m^3^) under RCP4.5 and RCP8.5. Every month’s section is a 1967–2100-time series of discharges. Black colours: transient simulations with climate changing and glacier retreat. Each point represents the runoff for each year, the curve shows the smoothed values. Blue colours: the same for idealized simulations with climate changing and glacier constant. Red colours: the same for idealized simulations with climate constant and glacier retreat. Graphical representation as in Guichard et al.^51^.

The relative influence of climate change and glacier retreat are analysed through a series of additional simulations (Fig. 4). They are designed to quantify the time component of the direct response of runoff to climate change (warming increases melting), and that of the indirect response (glaciers retreat, which decreases runoff). To that end, runoff is re-computed by relying on (i) evolving glaciers and a climate constant and (ii) constant glacier and a changing climate (Supplementary Methods Sect. 5). These analyses assess the relative importance of climate change, glacier retreat and also evapotranspiration increase for runoff changes (Fig. 4), and the time changes in the relative contribution of each of these parameters on overall discharge evolutions.

In summer (July, August and September) and under RCP4.5, transient runoff is mostly driven by climate change (direct warming effect) until 2020, and then glacier retreat has more influence until the end of the century. This confirms our first results concluding that, so far, glacier retreat has a relatively limited effect on water discharge. Same findings are highlighted under RCP8.5 in August and September. However, in June for example, runoff computed under constant climate is higher than the transient runoff for the late century, which happens also in June and July under the RCP8.5 scenario (Fig. 4). This is mostly due to sensibly modified evapotranspiration: under a constant climate, evapotranspiration remains stationary, which leads to a higher amount of water, by direct runoff coming from the non-glaciated parts of the catchment (the area of which increases in the future).

The significant influence of climate change in the beginning of the period and evapotranspiration thereafter is clearly highlighted by the analysis of relative weight, of the direct vs. indirect response of water discharge to climate change (Supplementary Results Sect. 6 and Supplementary Fig. S13). This estimation is obtained as the ratio between, on the one hand, the difference between runoff with glaciers constant and transient runoff, and in the other hand, the difference between runoff computed with climate constant and transient runoff. The role of the evapotranspiration is also clearly discernible when considering the monthly runoff for the not-glaciated parts of the catchment, for which runoff computed under a constant climate is higher than the transient runoff (Supplementary Fig. S12).

## Contribution of snow and ice melt to water discharge

Taken together, the results of Figs. 2 and 3 suggest (i) major changes in the hydrological regime of the Arve river, with discharge increase in winter and decrease in summer; and (ii) strong modifications in the relative contributions of snow and ice melt to total runoff.

**Figure 5:**
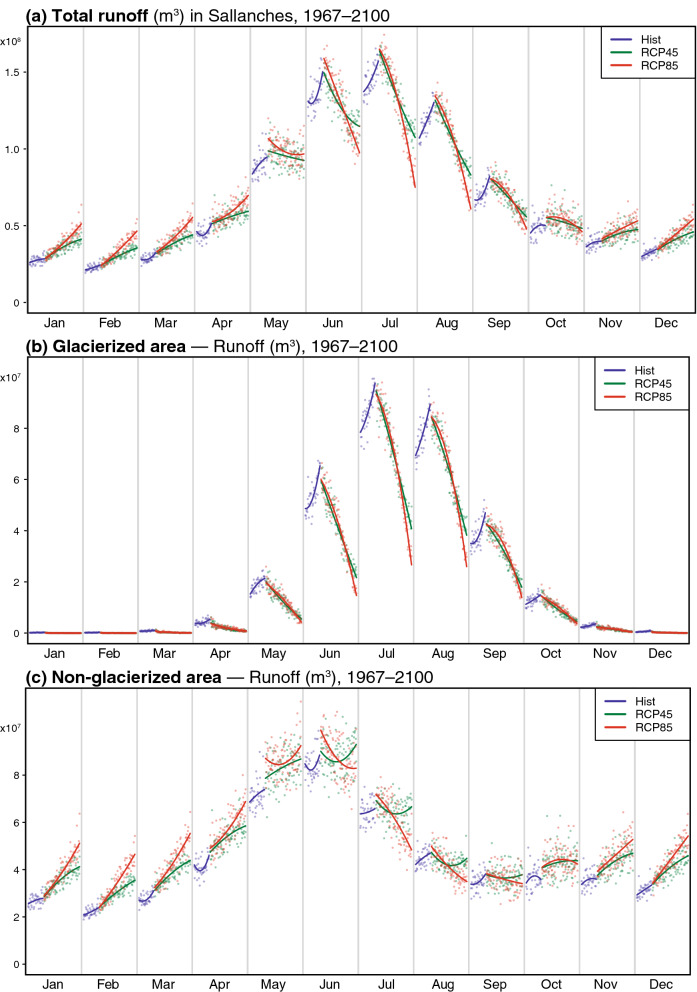
(**a**) Monthly total runoff in Sallanches (m^3^). (**b**) Monthly runoff of the ice-covered part of the catchment (m^3^). (**c**) Monthly runoff of the ice-free part of the catchment (m^3^). Every month’s section is a 1967–2100-time series of discharges. Blue colours: historical (Hist) simulations, period 1967–2018. Each point represents the runoff for each year, the curve shows the evolution trend with a quadratic model. Green colours: the same for the RCP4.5 simulations, period 2008–2100. Red colours: the same for the RCP8.5 simulations, period 2008–2100. Graphical representation as in Guichard et al.^51^.

Figure 5 represents projected changes in the hydrological regime, that is, changes in the annual cycle of runoff at the Sallanches station. Under current climate conditions, the Arve river in Sallanches has a clear nivo-glacial hydrological regime, characterized by a sharp increase in total runoff in spring due to snow melt, an annual peak in summer driven by ice melt, and very low runoff in winter (Fig. 5a). Throughout the century, the differences between winter and summer are simulated to continuously decrease. Wintertime runoff is expected to increase by 57 and 100% (for RCP4.5 and RCP8.5, respectively) between 2006 and 2100, with most runoff originating from the ice-free portion of the watershed (Fig. 5c). There are two main reasons explaining such an increase: a larger fraction of rainfall, and an increase in snowpack melting, even in the core of the winter season. In contrast, the summer season shows a dramatic drop in runoff by 30–45%, from the “peak water” reached nowadays to 2100 (Fig. 5a). This evolution is largely driven by the glacierized fraction of the watershed. A minor runoff decrease of 3–7% on the ice-free part (Fig. 5c) is associated with increased evapotranspiration from the non-glacierized surfaces (15–35%), and negative changes in summer precipitation (− 3 to − 23%). The major drop in water discharge from the glacierized part of the basin (− 50 to − 70%, Fig. 4b) is explained by the glacier retreat as modelled for the respective changes in climate forcing (Fig. 2e,f).

**Figure 6:**
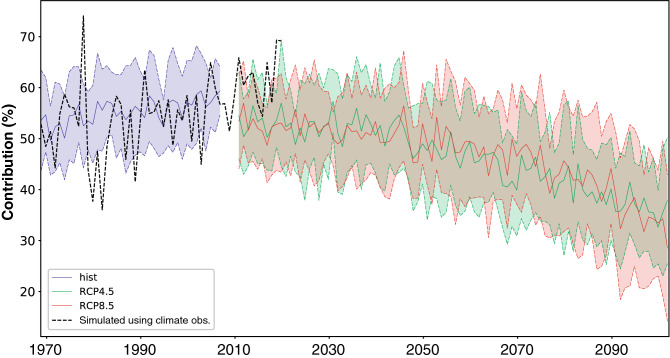
Relative contribution of the glacierized area to the total runoff (%) in summer (JJA). Black dotted curves: simulations using climate observations, period 1967–2018. Blue colours: historical (Hist) simulations, period 1967–2005. The solid curve shows the ensemble mean, the colour shading extend to ± 1 standard deviation to show model uncertainties. Green colours: the same for RCP4.5 simulations, period 2008–2100. Red colours: the same for RCP8.5 simulations, period 2008–2100.

The marked decrease in glacierized areas, associated with the strong increase in frontal glacier elevation (Fig. 2e,f, Supplementary Fig. S1), as well as the dramatic decrease in summertime water discharge throughout the century (Fig. 3e), raise the question of the evolution of the glacier contribution to the total water discharge for the Arve river. Ice melt contribution to the total runoff was already addressed in other regions such as High Asia, Chilean Andes and Italian Alps^37–39^. Forcing GSM-Socont with modelled historical climate indicates that the glacier contribution to the summer water discharge slightly increased in the last decades (Fig. 6), in coherence with the melt intensification favoured by ongoing warming (Figs. 2, 3). This increasing trend is sensibly stronger when the hydrological model is forced by climate observations, rather than historical simulations. This is more driven by precipitation than temperature. Summer precipitation amounts are larger in the observations than in the simulations and slightly increasing (Fig. 2d), causing snowfall at mid- and high elevations. This snow rapidly melts and enhances the contribution from the glacierized fraction of the watershed.

In the simulations forced by observed climate variables, the ice melt contribution was still increasing in recent years (Fig. 6). In those forced by climate models, in contrast, the contribution of glacierized areas reached an overall maximum in summer between the late twentieth and the early twenty-first centuries. The contribution peaked at 50–60% of the total summer discharge, and slightly decreased since then. This negative trend is expected to become more pronounced with time, with a decrease still accelerating during the second half of the century. By 2100, the annual contribution of the glacierized parts of the watershed could drop to 35–45%, these values being remarkably similar for both RCP4.5 and RCP8.5. In the meantime, the glacierized area is projected to drop from 85 km^2^ to 20–40 km^2^, with a marked difference between the two scenarios during the last decades of the century (Fig. 2e). Under RCP4.5 (RCP8.5) the total runoff decreases by 35% (40%) between 2006–2035 and 2071–2100, while runoff from the ice-covered part decreases by 45% (50%) (Fig. 5a, b). Thus, the ratio between the runoff from the entire catchment and the runoff from the ice-covered part is roughly the same for both RCPs. Similar evolutions of runoff under RCP4.5 and RCP8.5 are also linked more generally to the glacier evolutions. The latter are very similar under both RCPs in the first decades of the century, and are largely driven by the recent and present-day glacier geometry, because of the large inertia of glaciers, inducing a lag between temperature changes and their response^40^. A last explanation is that temperature and precipitation evolutions are quite similar under RCP4.5 and RCP8.5, with differences only appearing in the second part of the twenty-first century^17^.

Climate projections and associated impacts on the environment result from various processes that respond at different time scales, and that can combine or cancel out their specific effects. Better understanding these processes and their characteristic time steps is increasingly considered as a way to better analyze climatic projections^41,42^. In this study, this paradigm is applied to hydrological simulations in a partly glacierized catchment by distinguishing runoff evolution depending on climate change and glacier retreat. The effect of temperature increase and accelerated melting is of primary importance from 1965 to about 2020, while glacier retreat become predominant thereafter until about 2070. At the end of the century, enhanced evapotranspiration directly caused by warmer conditions, plays a major role on runoff evolution in summer, further accelerating runoff decrease in June under RCP4.5 and in June and July under RCP8.5. Direct impacts of climate change could be considered as a short term process responsible for the current or near-term “peak water” recorded in many alpine rivers and catchments. Indirect impacts of climate, through changes in the glacier extensions, become predominant for most future decades. Given the inertia of glaciers, including their area and frontal elevations, such indirect effect is expected to perturb peri-alpine hydrology for a long time, well beyond the end of the current century.

The evolution of the hydrological cycle detected with the simulations are in line with previous studies^25,26^. Changes in the runoff seasonality are depicted in other regions of the world^20,21,43^, such as in the South American Andes where, for example, runoff from the Juncal catchment, Chile, is expected to strongly decrease in summer under climate change and glacier retreat^44^. In addition to the assessments of the evolution of the hydrological cycle, the present study specifies the effects of glacier retreat and accelerated melting, and the balance between those two opposite effects. These processes, specific to partially-glacierized watersheds^45^, combine with the most general increase of evapotranspiration, that will increasingly act to decrease general runoff in many (but not all) regions of the world in future decades^46,47^.

This rapid change in the mountain cryosphere will lead to important shifts in the hydrological cycle. The accelerated melting of snow and ice under climate change leads to a temporal modification of the maximum discharge, with a gradual decrease of the summer flow. Our model results thus indicate a transition to a discharge regime that is more controlled by rainwater, rather than meltwater. This will have important repercussions on the seasonal availability of water, and consequently on the storage in and management of freshwater reservoirs. These changes may have impacts on hydropower production^48^, increase flood risk in winter and drought risk in summer^49^. Water temperature may also rise in summer, which could notably have impacts on ecosystems and for the cooling of nuclear power stations. All these effects could cause additional challenges managing hydropower and rivers.

## Methods

The choice of the conceptual reservoir-based model GSM-Socont is motivated by the low diversity of long meteorological^50^ series available in the studied watershed. The catchment is represented as a set of spatial units assumed to have a homogeneous hydrological behaviour. A first level of discretization is a separation between ice-covered and not ice-covered part in the catchment, and a second level of discretization consists in dividing those two parts into elevation bands. For each elevation band, precipitation and temperature time series are interpolated using vertical gradients. For the ice-covered part of the catchment, runoff is then computed two linear reservoir approaches for snow and for ice. For the ice-free part of the catchment, runoff is computed using a linear reservoir for the slow contribution of soil and underground water, and a non-linear reservoir for direct runoff. All those computation steps depend on seven equation parameters (Supplementary Table S2) that require calibration for each studied catchment. The calibration of the reservoir-based model GSM-Socont for the Arve catchment has been performed using observed precipitation, temperature and runoff, and detailed catchment characterization (Supplementary Methods Sects. 1 and 4). It produces hydrological discharge simulations at a daily timescale. The two first years of the simulation are discarded because they are affected by the model initialization. Simulations consisted in computing 1965–2018 runoff using observed climate, and then computing runoff from 1965 to 2100 using CMIP5 simulated climate. CMIP5 historical time series of precipitation and temperature were downscaled and bias-corrected using the CDFt method^35^. RCP4.5 and RCP8.5 were considered. Sixteen global climate models have been used to quantify model-dependency and related uncertainties (Supplementary Methods Sects. 2 and 3). During the historical period, the ice-covered area remains constant. Assuming an ice-cover decrease between 1965 and 2018 does not significantly influence water discharge (this hypothesis is tested in Supplementary Methods Sect. 4.3). For future simulations, glacier extension obtained from the GloGEMflow glaciological model^18^ are used to update the ice-covered area in 5-year steps until 2100. GloGEMflow consists of a surface mass balance component and an ice flow component which are combined to calculate the temporal evolution of every individual glacier. This glaciological model is extensively validated over the European Alps as (i) the modelled past glacier evolution coincides with observations on glacier geometry changes and surface velocities and (ii) the simulated future evolution is conform with results based on more complex 3D models^12^.

Projections of future runoff in a glacierized catchment have significant uncertainties due to the modelling process and uncertain evolution of climate and glacier retreat. Effects of erroneous calibration that can cause significant uncertainties in the modelling are unlikely, as calibration results show a good overall performance for the daily discharge simulation (Supplementary Results Sect. 1). Uncertainties in climate and glacier evolution refer to the input data (i.e. CMIP5 and GloGEMflow results) and are taken into account through the ensembles of model results presented here. Results on future runoff should thus be interpreted bearing in mind these uncertainties.

The experiment disentangling the effects of climate change and glacier retreat consists in modelling runoff either using climate changing and glacier constant or using constant climate and glacier retreat. GSM-Socont is thus run using either 2006-glacier extent or detrended climate as inputs (Supplementary Methods Sect. 5). Hydrological modelling and GSM-Socont calibration required powerful computer resources (Supplementary Methods Sect. 4).

## Supplementary information

Supplementary information

## Data Availability

The datasets generated during the current study are available from the corresponding author on a reasonable request.
