# Supplementary material for: The impact of climate change and glacier mass loss on the hydrology in the Mont-Blanc massif
Source: Sci Rep. 2020 Jun 26;10:10420. doi: 10.1038/s41598-020-67379-7 (PMC7319973; doi:10.1038/s41598-020-67379-7)
Supplement: Supplementary file 1 — Supplementary information [file 41598_2020_67379_MOESM1_ESM.docx]

The impact of climate change and glacier mass loss on the hydrology in the Mont-Blanc massif

Léa Laurent, Jean-François Buoncristiani, Benjamin Pohl, Harry Zekollari, Daniel Farinotti, Matthias Huss, Jean-Louis Mugnier, Julien Pergaud

**Supplementary Information**

# Supplementary Methods

## 1. Data collection

In this study, we use (1) climate and hydrological data observed by government agencies and (2) glacier evolution data derived from models. For climate data, observed minimum temperature (Tn, °C), maximum temperature (Tx, °C) and precipitation (P, mm) were extracted at a daily timescale between 1965 and 2018 from the Météo-France weather station network. Climate data are measured with Météo-France weather station equipment^1^. The daily mean temperature T2 (°C) was computed as T2 = (Tn+Tx)/2, and potential evapotranspiration (ET_0_) was estimated using the Thornthwaite formula^2^. For the runoff of the Arve river, we used observed daily discharge (m^3^/s) at the Sallanches outlet from 1965 to 2018 as provided by the Banque Hydro France. Daily discharge is the mean of all the discharges on a given day. The record is slightly perturbed by the presence of the Emosson dam at the border between France and Switzerland: this perturbation is intermittent, and reaches at most 10% of the total runoff recorded in Sallanches. The relative perturbation is much larger closer to Chamonix, up to 30% at the “Pont des Favrands” outlet, hereby explaining why this second outlet was not considered.


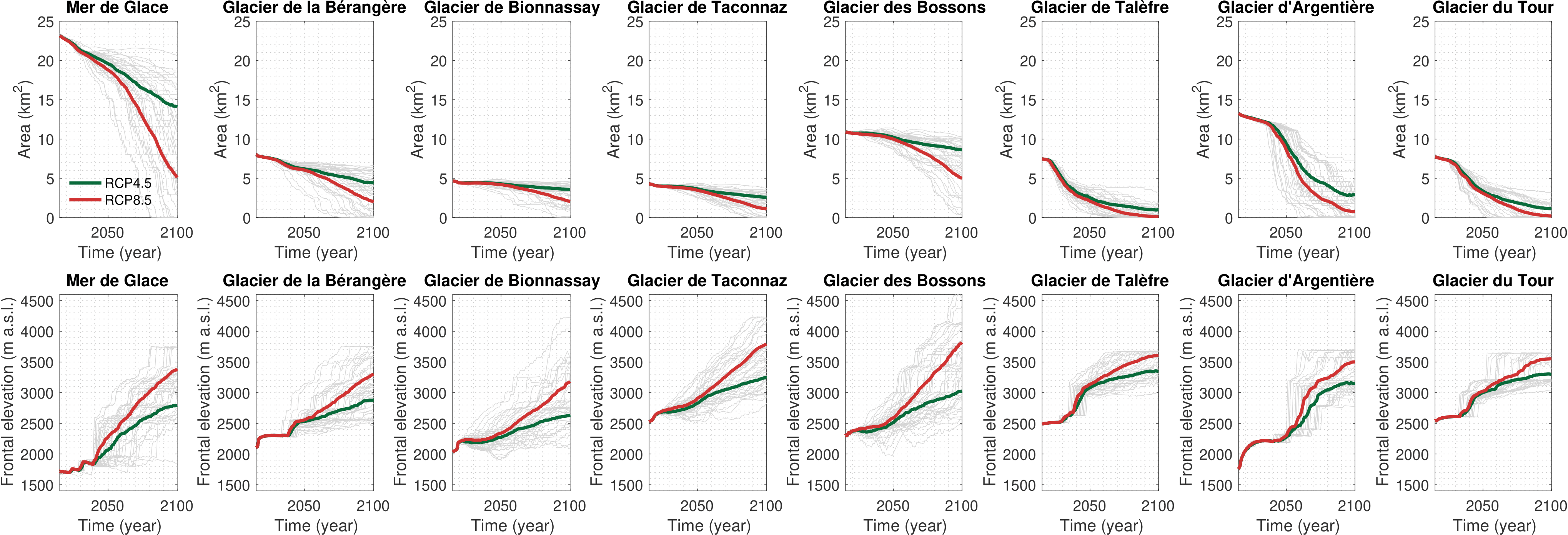


**Supplementary Figure S1 |** Changes in glacier area and frontal elevation for glaciers in the Mont Blanc Massif between 2015 and 2100. The bold coloured lines correspond to the multi-simulation mean, while the thin grey lines represent the individual simulations (RCP4.5 and RCP8.5). Individual simulations correspond to the glacier evolution modelled with GloGEMflow under different RCM simulations from the EURO-CORDEX ensemble. For more information concerning the glacier model and the simulations, refer to Zekollari et al. (2019)^3^.

The 2006-2100 evolutions of the areas and frontal elevations of the 8 studied glaciers under RCP4.5 and RCP8.5 are derived from Zekollari et al.^3^ (**Supp. Fig. S1**). These data are computed using the GloGEMflow glaciological model, which consists of a surface mass balance component and an ice flow component that are combined to calculate the glacier’s temporal evolution. The model explicitly accounts for ice dynamics, which is generally neglected in hydrological models^4,5^. The glacier evolution is calculated from high-resolution RCM output, downscaled from GCM output. This contrasts with the climatological data used in this study, which come from bias-corrected and statistically downscaled GCMs. The modelled future glacier evolution is however strongly driven by the RCP and results obtained when forcing the glacier model with RCM data (in Zekollari et al., 2019)^3^ vs. GCM data directly (in Marzeion et al., 2020)^6^ were shown to be very similar.

## 2. CMIP5 Earth System Models

In this paper, 16 global climate models are selected among the 39 reported in the Fifth Assessment Report of the IPCC^7^. The outputs of these models are used as climate inputs to the hydrological model, and the selection is based on the availability of the requested simulations (historical, RCP4.5 and RCP8.5) and variables (daily Tn, Tx and precipitation). All data are available at daily timescale. The 16 models and the institution in charge of the models developments are listed here:

- ACCESS1-0 and ACCESS1-3: CSIRO (Commonwealth Scientific and Industrial Research Organisation, Australia), and BOM (Bureau of Meteorology, Australia), Australia
- CanESM2: Canadian Centre for Climate Modelling and Analysis, Canada
- CNRM: Centre National de Recherches Météorologiques / Centre Européen de Recherche et Formation Avancées en Calcul Scientifique, France
- GFDLCM3, GFDLM2G, GFDLM2M: Geophysical Fluid Dynamics Laboratory, USA
- INMCM: Institute for Numerical Mathematics, Russia
- IPSL-CM5A-LR, IPSL-CM5A-MR, IPSL-CM5B-LR: Institut Pierre-Simon Laplace, France
- MIROC, MIROC5: Atmosphere and Ocean Research Institute (The University of Tokyo), National Institute for Environmental Studies, and Japan Agency for Marine-Earth Science and Technology, Japan
- MPI-LR, MPI-MR: Max Planck Institute for Meteorology (MPI-M), Germany
- MRI: Meteorological Research Institute, Japan

The RCP4.5 forcing is a rather optimistic scenario while RCP8.5 forcing is the most pessimistic one. The climate data are extracted at the nearest grid point of the Chamonix station using historical model runs for the period 1965-2005 (that is the period for which daily river discharge records are available), and whilst RCP simulations for 2006-2100.

## 3. CDFt downscaling: general principles and application to CMIP5 simulations

For impact studies, such as glacier hydrology, it is necessary to correct climate model outputs and remove their bias. To do this, the Cumulative Distribution Function method (CDFt)^8,9^ is used. It basically consists in a statistical downscaling procedure using the cumulative density functions (CDF) of the modelled and observed variables. The first step is to find a relationship between the CDF of the data observed at the weather station and the CDF of the modelled data (at coarse resolutions, and over the same period). This relationship is a transfer function. Then, assuming persistency, the same transfer function is applied to adjust the CFD of the future modelled data. These refinements allow for a reduction of biases but perturb the energy, mass and momentum conservations in the model outputs. In this study, the CDFt method is independently applied to daily Tn, Tx and P. The so corrected variables are then used for computing the daily mean temperature and ET_0_.

## 4. GSM-Socont hydrological model

### 4.1 Model presentation

GSM-Socont is a conceptual reservoir-based model having the mean temperature T2 ($T2= \frac{T_{min}+T_{max}}{2}$), precipitation P and potential evapotranspiration ET_0_^10^ as input variables. The model runs with daily resolution. The choice of this relatively simple model is motivated by the scarcity of meteorological data available in the analysed watershed. In addition, simpler hydrological models are preferable in the analysis of the hydrological responses of a watershed in the long term, as they were shown to optimally predict hydrological variables^11^.

The catchment is represented as a set of spatial units, assumed to have a homogeneous hydrological behaviour. The model has two levels of discretization. The first level corresponds to the separation between ice-covered and not ice-covered part in the catchment. Each of these areas is characterized by its surface and its hypsometry. The second level of discretization consists in dividing the ice-free and glacierized parts into elevation bands (we choose a distance of 250m in elevation bands). For each band, precipitation and temperature time series are interpolated according to their mean elevations using constant vertical gradients.

Water runoff is computed separately for each band, and for ice-covered and ice-free areas. For the total catchment, the mean specific runoff $Q$ (mm.d^-1^) on a given day is given by:

$$Q= \frac{1}{a_{c}}\times\sum_{i=1}^{2} \sum_{j=1}^{n_{i}} a_{i,j} \times Q_{i,j}$$

where $i$ is an index for each of the two parts of the catchment an $j$ an index for each of the $n_{i}$elevation bands in part $i$. $a_{i,j}$ (km²) is the area of an elevation band $j$ belonging to the catchment part $i$, and $Q_{i,j}$ (mm.d^-1^) is the mean specific runoff from this spatial unit. $a_{c}$ (km²) is the area of the entire catchment.

For each elevation band, the temporal evolution of the snow pack is computed through an accumulation and melt model. The state of precipitation (solid or liquid) is determined by a temperature threshold:

$$P_{snow}= P_{tot}, P_{liq}=0, T\leq T_{0}$$

$$P_{snow}=0, P_{liq}=P_{tot}, T\geq T_{0}$$

where $P_{tot}$ (mm.d^-1^) is the total precipitation on a given day, $P_{snow}$ (mm.d^-1^) is the solid and $P_{liq}$ (mm.d^-1^) is the liquid precipitation. $T$ (°C) is the mean daily air temperature and $T_{0}$ (°C) is a threshold temperature, set to 1°C in this study with a temperature interval of 2°C in which transition between snow and rain occurs. The potential snowmelt $M_{p,snow}$ (mm.d^-1^) is computed with a degree-day approach :

$$M_{p,snow}=\left\{ \begin{aligned} a_{snow}(T-T_{m}), &T>T_{m} \\ 0, &T<T_{m} \end{aligned} \right.$$

where $a_{snow}$ is the degree-day factor for snowmelt (mm.d^-1^.°C^-1^) and $T_{m}$ the threshold temperature for melting, set to 0°C. The actual snowmelt $M_{snow}$ (mm.d^-1^) is computed depending on the available snow height $H_{s}$ (mm water equivalent). This degree-day approach is also used for the ice melt computation, replacing all subscripts “snow” by “ice” in the equation. The snow and ice melt computation requires to calibrate two parameters: the degree-day coefficients for snow $a_{snow}$ and ice $a_{ice}$.

For the ice-covered part of the catchment, the runoff model consists of a simple linear reservoir approach^12^. Two parallel linear reservoirs are used, one for snow and one for ice. Snow and ice melt are turned into runoff by linking the water volume of each reservoir to the runoff with a coefficient that needs to be calibrated ($k_{ice}$ and $k_{snow}$). We assume that ice melts only when all snow of a given elevation band has disappeared. Total runoff of the ice-covered area is the sum of snow and ice melt ($Q_{snow}+Q_{ice}$, m^3^.s^-1^). For the ice-free part of the catchment, the model computes an equivalent rainfall (mm.d^-1^) corresponding to the sum of liquid precipitation and snowmelt for each elevation band. The equivalent rainfall-runoff transformation is carried out through a conceptual reservoir-based model^13^. It is composed of a linear reservoir for the slow contribution of soil and underground water, and a non-linear reservoir for direct runoff. The slow reservoir has two possible outflows, the base flow and the actual evapotranspiration. The effective rainfall as well as the actual evapotranspiration are conditioned by the filling ratio $S_{slow}/A$ of the slow reservoir, where $S_{slow}$ (mm) is the actual storage and $A$ (mm) is its maximum storage capacity. The base flow $Q_{base}$(m^3^.s^-1^) is related linearly to the actual storage through the reservoir coefficient $k_{slow}$. The quick-flow component $Q_{quick}$ (m^3^.s^-1^) is modelled through a non-linear storage-discharge relationship $Q_{quick}= \beta\times J^{\frac{1}{2}}\times H^{\frac{5}{3}}$ where $J$ is the slope of the catchment, $H$ the actual storage of the reservoir and the parameter $\beta$ requires calibration. The total runoff from the not ice-covered part of the catchment corresponds to the sum of the quick and the base flows. The mean specific runoff for each elevation band is the sum of their ice-covered and ice-free parts: $Q_{i,j}= Q_{snow}+ Q_{ice}+ Q_{base}+ Q_{quick}$.

Overall, GSM-Socont has 7 parameters to calibrate: $a_{snow}$, $a_{ice}$, $k_{snow}$, $k_{ice}$, $A$, $k_{slow}$ and $\beta$. The calibration procedure is detailed in the next section.

A file with detailed characterization of the catchment and settings is also needed and contains:

- The temperature lapse rate (–0,57°C/100m), obtained from a linear regression of mean daily temperature observations from the Chamonix (1042m), Sallanches (541m) and Aiguille du Midi (3845m) meteorological stations;
- The precipitation gradient, interpolated with a linear regression using daily precipitation recorded at Chamonix and Sallanches, and equal to + 2,7%/100m;
- The threshold temperature between rain and snow, set to 1°C^10^;
- The temperature interval in which transition between rain and snow occurs, set to 2°C^10^;
- The elevation of the weather station where the daily T2 and P amount were measured, equals here to 1042m for Chamonix station;
- The total catchment basin area (658km^2^ here);
- The present proportion of ice-covered part of the catchment basin (16%);
- The mean slope of the ice-covered part of the catchment basin (0.57);
- The number of elevation bands used for the ice-covered part of the catchment (15 here);
- The number of elevation bands used for the non-ice-covered part of the catchment (62).

Two hypsometric information for each of the two parts of the basin (ice-covered and ice-free) is needed as input as well. The spatial discretization is based on a digital elevation model with a resolution of 25m (IGN). Glacier outlines are taken from the Randolph Glacier Inventory^14^.

### 4.2. Model calibration

The model has been calibrated using observed meteorological data from 01/01/1987 to 31/12/2001, that is the longest availability period of both hydrological and meteorological records. Outflows are also simulated for the period 01/01/2010 to 31/12/2017, for which observations are available to validate the model (observations during that period were not used for model calibration). For all simulations, the first two years are discarded since they correspond to the spin-up of the model.

An initial parameter set is chosen among 10,000 randomly generated sets^10^. Criteria used to select this set are the bias between simulated and observed discharge $V_{ed}$, the Nash criterion $Nash$, and a Nash criterion calculated on the log values of the discharges ${Nash}_{log}$:

$$V_{ed}= \sum_{i=1}^{n} (Q_{mes,i}-Q_{sim,i})\times({\sum_{i=1}^{n} Q_{mes,i})}^{-1}$$

$$Nash=1-\sum_{i=1}^{n} \left( Q_{mes,i}-Q_{sim,i} \right)^{2}\times{(\sum_{i=1}^{n} (Q_{mes,i}-\bar{Q_{mes}})^{2})}^{-1}$$

$${Nash}_{log}=1-\sum_{i=1}^{n} {(\ln\left( Q_{mes,i} \right)-\ln(Q_{sim,i}))}^{2}\times{(\sum_{i=1}^{n} (\ln\left( Q_{mes,i} \right)-\frac{1}{n}\sum_{j=1}^{n} \ln(Q_{mes,j}))^{2})}^{-1}$$

where $Q_{mes,i}$ is the observed discharge and $Q_{sim,i}$ is the simulated discharge on day $i$. $n$ is the number of days of the simulation period. The bias $V_{ed}$ provides information about the error between observed and modelled runoff. Nash criterion informs about the quality of the simulation during peak runoff, especially during the ablation season, and Nash_log_ criterion informs about the quality of the simulation of the base flow^15^. Nash and Nash_log_ criteria vary between $-\infty$ and 1, and tend towards 1 when the simulation get close to reality. The choice of the initial set according to these criteria is based on the following steps: i) we retain all parameter sets with $V_{ed}$<0,02; ii) we then retain the 1% best sets according to the Nash criterion; and iii) the 1% best sets according to the Nash_log_ criterion. If more than one set is retained, we reiterate by lowering the thresholds for steps ii) and iii). For the random generation, the parameters are supposed to be uniformly distributed within an interval based on the results of case studies (given in **Supp. Table S2**).

| Parameter | Unit | Min. value | Max. value | Reference |
| --- | --- | --- | --- | --- |
| $\boldsymbol{a}_{\boldsymbol{ice}}$ | mm.d^-1^.°C^-1^ | 5,0 | 20,0 | Rango et Martinec, 1995^16^; Singh et al., 2000^17^; Hock, 2003^18^ |
| $\boldsymbol{a}_{\boldsymbol{snow}}$ | mm.d^-1^.°C^-1^ | 1,3 | 11,6 |  |
| $\boldsymbol{k}_{\boldsymbol{ice}}$ | d | 0,2 | 15,0 | Baker et al., 1982^12^; Klok et al., 2001^19^ |
| $\boldsymbol{k}_{\boldsymbol{snow}}$ | d | 4,0 | 18,0 | Baker et al., 1982^12^; Klok et al., 2001^19^ |
| $\boldsymbol{A}$ | mm | 10 | 3000 | Consuegra et al., 1998^20^; Guex et al., 2002^21^ |
| $\mathbf{log}\mathbf{}\boldsymbol{(}\boldsymbol{k}\boldsymbol{)}$ | log(1/h) | -12 | -2 | Consuegra et al., 1998^20^; Guex et al., 2002^21^ |
| $\boldsymbol{\beta}$ | m^4/3^.s^-1^ | 100 | 30 000 | Consuegra et al., 1998^20^; Guex et al., 2002^21^ |

**Supplementary Table S2 |** Parameter intervals used for random generation and reference case studies.

Based on this first-guess, all parameters are optimized by varying one or two of them (regarding their role in the simulation) while keeping the others constant. For each parameter, or couple of parameters, the optimization criteria defined in Schaefli et al 2005 are used. The order of fine-tuning is motivated by the model sensitivity to the 7 model parameters.

- $a_{snow}$ and $a_{ice}$ determine melting and have therefore a large influence on modelled outflows: the mean annual discharge bias ($V_{ed}$) is used as an objective function for their identification. At second order, their choice can be based on an additional metric, the $Nash$ criterion – with always $a_{ice}$>$a_{snow}$^22^;
- $A$ and $k_{slow}$ influence the base flow: the ${Nash}_{log}$ criterion is the most appropriate here;
- $k_{snow}$ and $k_{ice}$ are optimized using the $Nash$ criterion calculated for the period of snow and ice melt (15 July to 15 September) – with always $k_{ice}$<$k_{snow}$^23^;
- $\beta$ influences the skill during precipitation events: optimization is based on the $Nash$ criterion calculated over all days that satisfy the following conditions i) the ratio between the maximum discharge and the minimum discharge observed during the 3 days period including the preceding, the current and the following day, is higher than 1.5 and ii) the total rainfall over the same period is higher than 10mm.

Couples of parameters are calibrated by varying their values within a smaller interval than that used for the random set. As an example, values of the $a_{snow}$ and $a_{ice}$ couple are approached to the unit by computing statistical criteria for several simulations in which $a_{snow}\in[1:20]$ and $a_{ice}\in[1:20]$ and vary with a step of 1. This represents 20x20 = 400 model runs for all the years chosen for the calibration period. Then the couple values are refined using a smaller step (here 0.01). This method is repeated for each couple of values mentioned above.

### 4.3. Outflow modelling

After model calibration, parameters are used to simulate runoff at the Sallanches outlet, for both historical and future periods. In a first step, a reconstructed runoff based on climate observations is obtained using Météo-France data from 1965 to 2018. The ice-covered part of the catchment is considered as constant. To test the impact of this assumption, two outflow series are simulated between 1965 and 2018: one using the 1967 glacier extent and one using the extent of 2004^14^. The root-mean-square error (RMSE) between both series equals 0.45% of the total runoff, suggesting that the effect of the constant-glaciers assumption is negligible. This implies that, over the recent decades, the decrease in ice-cover did not significantly modify water discharge as recorded at the Sallanches outlet. Hence, glacier outlines are set to their 2004 extents for all past simulations.

In a second step, the discharge is modelled from 1965 to 2100 using CMIP5 climate forcing under RCP4.5 and RCP8.5. The ice-covered area is expected to strongly decrease until 2100 and (**Supp. Fig. S1**), to take this effect into account, the ice-cover is updated in the hydrological model every 5 years (**Supp. Methods Section 1**).

## 5. Disentangling climate change and glacier retreat effects

Idealized simulations are designed to quantify the characteristic time response of the direct response of runoff to climate change (warming enhancing melting processes), and that of the indirect response (glaciers retreat, which decreases runoff in return). The numerical experiments consist in modelling runoff either using climate changing and glacier constant or using constant climate and glacier retreat.

The first part of the experiment consists in running GSM-Socont using CMIP5 climate variables under both scenarios RCP4.5 and RCP8.5. and 2006 glacier extent as inputs. Thus, simulated runoff assesses the direct effect of climate change on melting processes.

The second part of the experiment consists in running GSM-Socont using evolving glacier extent under both scenarios RCP4.5 and RCP8.5 – as it is done in the transient simulations (**Supp. Methods Section 4.3.**) – and constant climate inputs. To that end, low-frequency variability in the CMIP5 climate variables, i.e. temperature and precipitation, is first filtered out to remove all long-term trends to the data. Evapotranspiration is then re-calculated using these detrended series. Thus, simulated runoff assesses the direct effect of glacier retreat on melting processes.

# Supplementary Results

## 1. Calibration Results

**Supp. Table S3** presents the 7 optimal parameters retained after the calibration discussed above.

| $\boldsymbol{a}_{\boldsymbol{ice}}$ | $\boldsymbol{a}_{\boldsymbol{snow}}$ | $\log\boldsymbol{k}_{\boldsymbol{slow}}$ | $\boldsymbol{A}$ | $\beta$ | $\boldsymbol{k}_{\boldsymbol{ice}}$ | $\boldsymbol{k}_{\boldsymbol{snow}}$ |
| --- | --- | --- | --- | --- | --- | --- |
| 5 | 4,8 | -8,4 | 487 | 8080 | 1,1 | 4 |

**Supplementary Table S3 |** Model coefficients retained in this study for the Sallanches catchment.

| **Calibration period** | $\boldsymbol{R}^{2}$ | $\boldsymbol{V}_{\boldsymbol{ed}}$ | ***Nash*** | $\boldsymbol{Nash}_{\boldsymbol{log}}$ |
| --- | --- | --- | --- | --- |
| **1 Jan 1989-12 Dec 2001** | 0,92 | 0,012 | 0,81 | 0,86 |
| **Evaluation period** | $\boldsymbol{R}^{2}$ | $\boldsymbol{V}_{\boldsymbol{ed}}$ | ***Nash*** | $\boldsymbol{Nash}_{\boldsymbol{log}}$ |
| **1 Jan 2012-31 Dec 2017** | 0,92 | 0,00062 | 0,84 | 0,86 |

**Supplementary Table S4 |** Model evaluation.

**Supp. Table S4** shows the skill scores computed for the calibration and the validation periods. These scores are within the interval given in **Supp. Table S2**), with $V_{ed}<0,02$, and the two Nash criteria close to 1. The same conclusion is reached for the evaluation period. The GSM-Socont hydrological model shows a good overall performance for the daily discharge simulation over the calibration and the validation period (**Supp. Table S4**). Thus, the model reproduces well the observed outflow (**Supp. Fig. S5**).


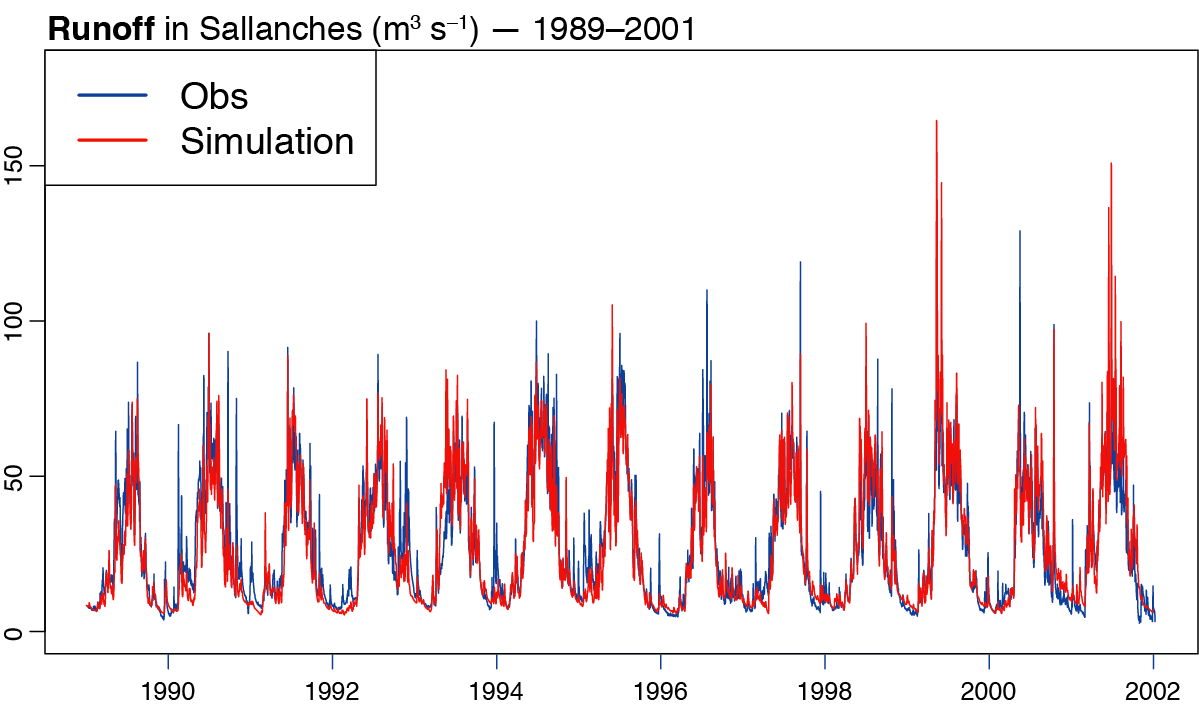


**Supplementary Figure S5 |** Daily runoff in Sallanches (m^3^.s^-1^), period 1989-2001. Blue curve: observed runoff. Red curve: simulated runoff.

| Coefficients | Spring | Summer | Autumn | Winter |
| --- | --- | --- | --- | --- |
| Pearson’s correlation coefficient (inter-annual variability) | 0,82 | 0,53 | 0,77 | 0,58 |
| Linear regression coefficient (low-frequency trends) | 0,81 | 0,47 | 0,75 | 0,52 |

**Supplementary Table S6 |** Seasonal calibration period coefficients depicting the quality of the simulation

The root-mean-square error between observed and simulated runoff is about 30% of the mean observed runoff over the calibration period. The coefficient of correlation is around 0.84, which denotes a strong co-variability between observed and simulated runoff. **Supp. Table S6** shows a good Pearson’s correlation coefficient between annual mean simulated and observed runoff in summer, inter-annual variability is thus well reproduced in this season. The linear regression coefficient between the same data suggests that summer trends are thus under-estimated during the calibration period. The quality of the simulation of the annual variability and trend is the best in spring, when Pearson’s correlation coefficient and linear regression coefficient are the highest. **Supp. Fig. S5** shows the good fit between the observed and simulated time series during the calibration period, despite some peak flows that are not well reproduced, in winter for instance.

## 2. Contribution of the ice-covered part of the catchment to the total outflow


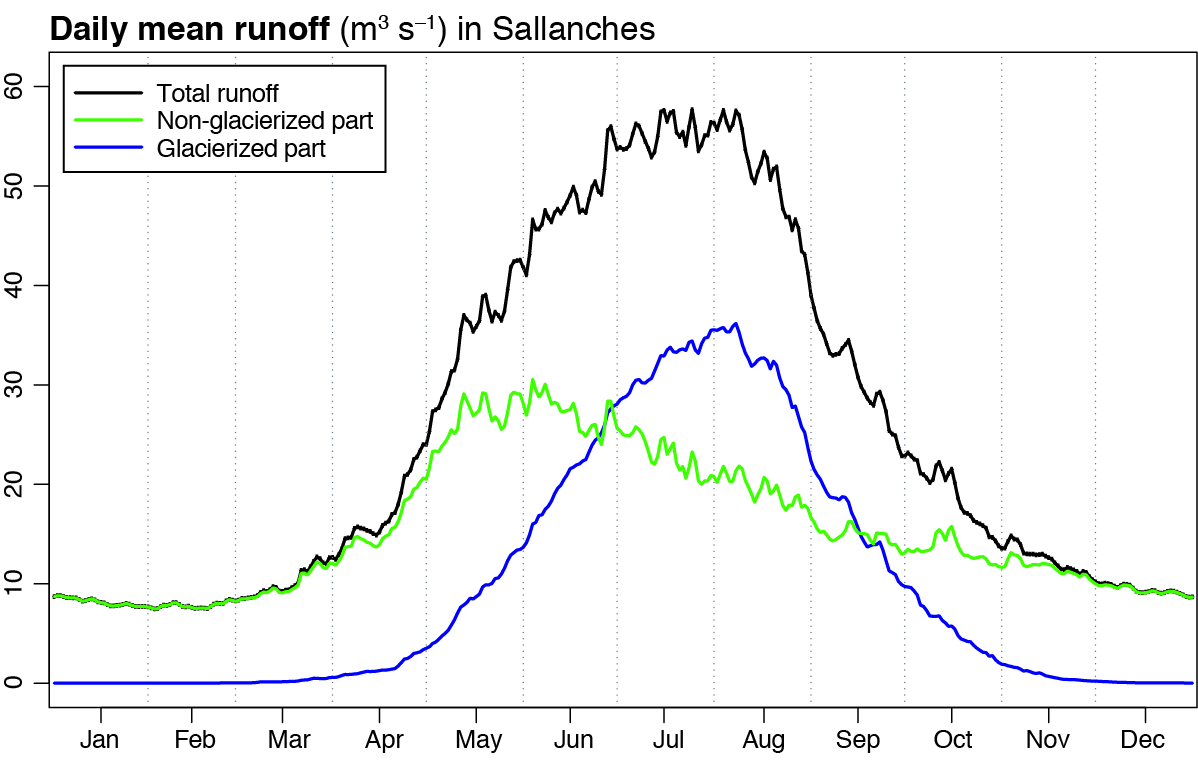


**Supplementary Figure S7 |** Daily mean runoff in Sallanches (m^3^.s^-1^), period 1967-2018.

The hydrological regime of the Arve river in Sallanches is typical of mid-latitude glacier rivers (**Supp. Fig. S7**). In winter, runoff is stationary and quite low (10m^3^.s^-1^). It starts to increase in April during the transition period of boreal spring. From June to September outflows reach 60m^3^.s^-1^, during the glaciers ablation period. Autumn is a second transition period with runoff decreasing back to its low water (winter) level.

Separating runoff from the ice-covered part and ice-free parts of the watershed shows that their relative contributions are highly variables throughout the year (**Supp. Fig. S7**). Runoff from the ice-free part is the only water supply to the total runoff in winter (Dec, Jan, Feb) due to the snow accumulation on the ice-covered part. It is still preponderant in spring (Mar, Apr, May: 75% in May), but the contribution of the ice-covered part increases in the meantime. The latter becomes dominant in summer (Jun, Jul, Aug: 60% in July and August) due to the maximum annual of snow- and then ice-melting. Then, in autumn (Sep, Oct, Nov), not-ice-covered part becomes the main contributor to the total runoff again when air temperature decrease driving snow and ice stopping melting.

## 3. Runoff variability

Simulated runoff is presented in the main paper (**Fig. 5**). In order to evaluate the time evolution of runoff day-to-day variability (in the context of a strongly changing average), **Supp. Fig. S8** presents the daily standard deviation of the runoff forced with CMIP5 climate data from 1965 to 2100.

**
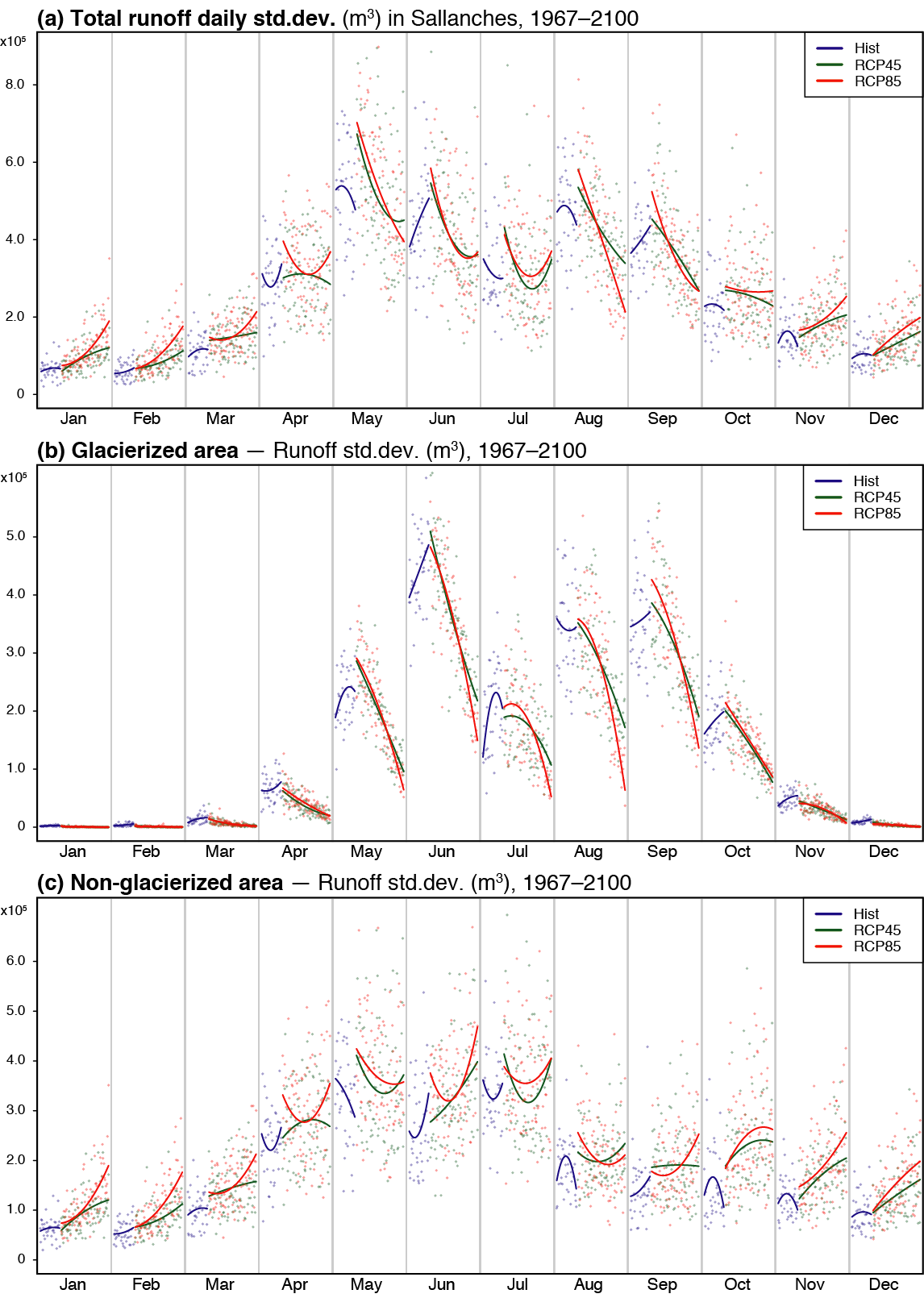
**

**Supplementary Figure S8 | (a)** Daily standard deviation of the total runoff in Sallanches (m^3^). **(b)** The same for the ice-covered part of the catchment (m^3^). **(c)** The same for the ice-free part (m^3^). Every month’s section is a 1967-2100-time series of standard deviation. Blue colours: historical (Hist) simulations, period 1967-2018. Each point represents the day-to-day standard deviation for each month of each year, the curve shows the evolution trend smoothed with a quadratic model. Green colours: the same for the RCP4.5 simulations, period 2008-2100. Red colours: the same for the RCP8.5 simulations, period 2008-2100.

The standard deviation of the total daily runoff increases in winter and strongly decreases in summer over the century (**Supp. Fig. S8a**). Standard deviation decreases from 6x10^5^m^3^ to 2x10^5^m^3^ in August under RCP8.5. Runoff variability could vary therefore the same way as its average by 2100. Runoff variability from the ice-covered part significantly decreases as well, especially from May to September (**Supp. Fig. S8b**). It could be due to the large decrease of the ice-covered area itself, involving a decrease of the runoff coming from this part of the catchment. In contrast, runoff variability from the non-glacierized part slightly increases from January to December in the future (**Supp. Fig. S8c**), which could be linked to associated area, and/or to an increasing proportion of liquid precipitation in winter, causing rapid discharge. Changes in the standard deviation could be also explained by the 0-bounded distribution, its values increasing thus when the average increases. Another hypothesis involves a change in the standard deviation of climate variables influencing runoff in the future. These hypotheses are tested below.

## 4. Temperature and precipitation variability

Evolutions of mean seasonal temperature and cumulative seasonal precipitation are presented in the main paper in **Fig. 2**. Seasonal standard deviation of precipitations and temperatures (winter and summer) are shown in **Supp. Fig. S9** to test the hypotheses mentioned above.


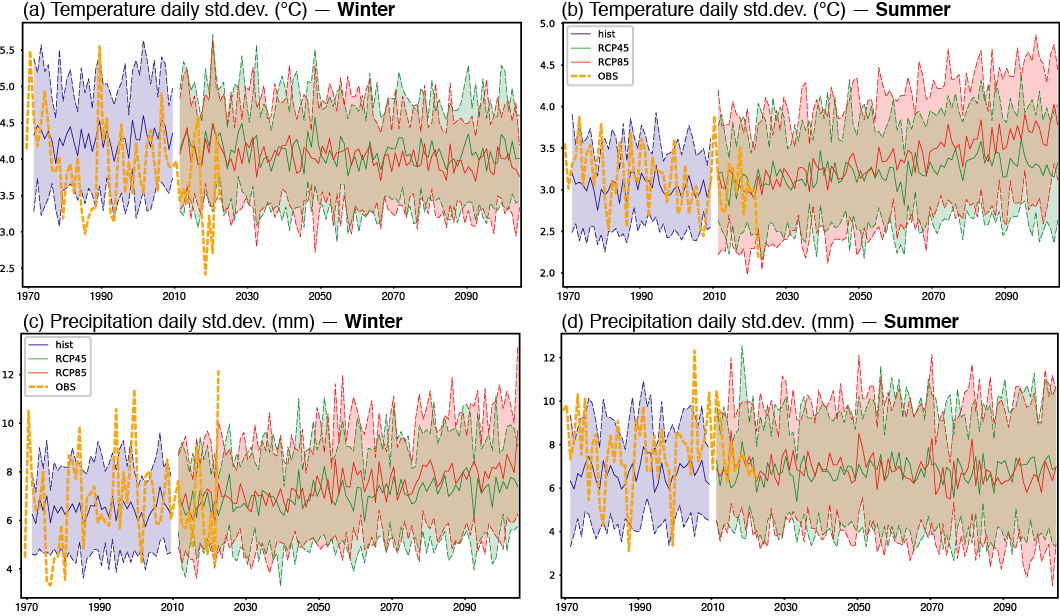


**Supplementary Figure S9 | (a)(b)** Daily standard deviation of winter and summer temperatures (°C). **(c)(d)** Daily standard deviation of winter and in summer precipitations (mm). Yellow curves: observations at the Chamonix station, period 1965-2018. Blue colours: historical (Hist) simulations, period 1967-2005. The solid curve shows the ensemble mean, the colour shading extends to ± 1 standard deviation to show model uncertainties. Green colours: the same for RCP4.5 simulations, period 2008-2100. Red colours: the same for RCP8.5 simulations, period 2008-2100.

Standard deviations of the precipitation amounts slightly increase in winter and slightly decrease in summer (**Supp. Fig. S9c, d**), following the evolution of cumulative precipitations (**Fig. 2**). Precipitations being bounded by 0 and discrete in time and space, are therefore very sensitive to mean changes. Standard deviations of temperatures slightly decrease in winter but increase more significantly in summer (**Supp. Fig. S9a, b**). Standard deviation is around 3°C in 2006 and reaches nearly 4°C in 2100 under RCP8.5. Temperature variability could therefore increase in summer by 2100. Standard deviations of the temperatures and of the runoff have opposite trends in summer and in winter (**Supp. Fig.** **S8a** and **Supp. Fig. S9a, b**). Standard deviations of the precipitations present weaker changes than standard deviations of runoff (**Supp. Fig.** **S8a** and **Supp. Fig. S9c, d**). Thus, temperature and precipitation variability cannot explain changes in runoff standard deviation, which leads to retain the second hypothesis: evolution of runoff standard deviation and variability should be primarily related to changes in the mean values.

## 5. Runoff future evolution under constant glacier extent

Future runoff is simulated using a variable ice-covered area. In order to show how glacier retreat affects the results and thus the estimated runoff evolution for the future, idealized simulations are performed using RCP4.5 and RCP8.5 climate forcing but with constant glacier areas, set to their observed 2006 and estimated 2095 extensions as obtained by Zekollari et al.^3^. Results are shown in **Supp. Fig. S10** and **Supp. Fig.** **S11**.

Runoff mainly changes in summer under a 2006 constant glacier area (**Supp. Fig. S10a** and **Supp. Fig11a**). Total runoff increases from 1.5x10^8^ to nearly 2.0x10^8^m^3^ in July under RCP4.5, and up to 2.3x10^8^m^3^ under RCP8.5. Under a 2095 constant glacier area and RCP4.5 forcing, runoff also slightly increase (**Supp. Fig. S10a**). Temperatures are likely to rise significantly from 2006 to 2100 under the two RCP forcing, increasing gradually snow and then ice melt (**Fig. 2**). Thus, runoff could rise significantly, with a change rate logically much larger under large ice-covered fractions (that is, under the 2006 glacier extension conditions). Under a much-reduced ice-covered area (2095 glacier extension), snow and ice melt discharge is much lower too, and runoff changes are weaker as well. Total runoff is even projected to decrease under a RCP8.5 forcing and a 2095 constant glacier area in summer (**Supp. Fig. S11a**). The important warming occurring under this scenario could lead to a strong increase of evapotranspiration over most parts of the watershed, especially the ice-free, lower and thus warmer parts. It could also cause a decrease of snow amounts on the catchment, even at high elevations experiencing much more frequent thaw events^24^, acting thus to reduce snow melt.


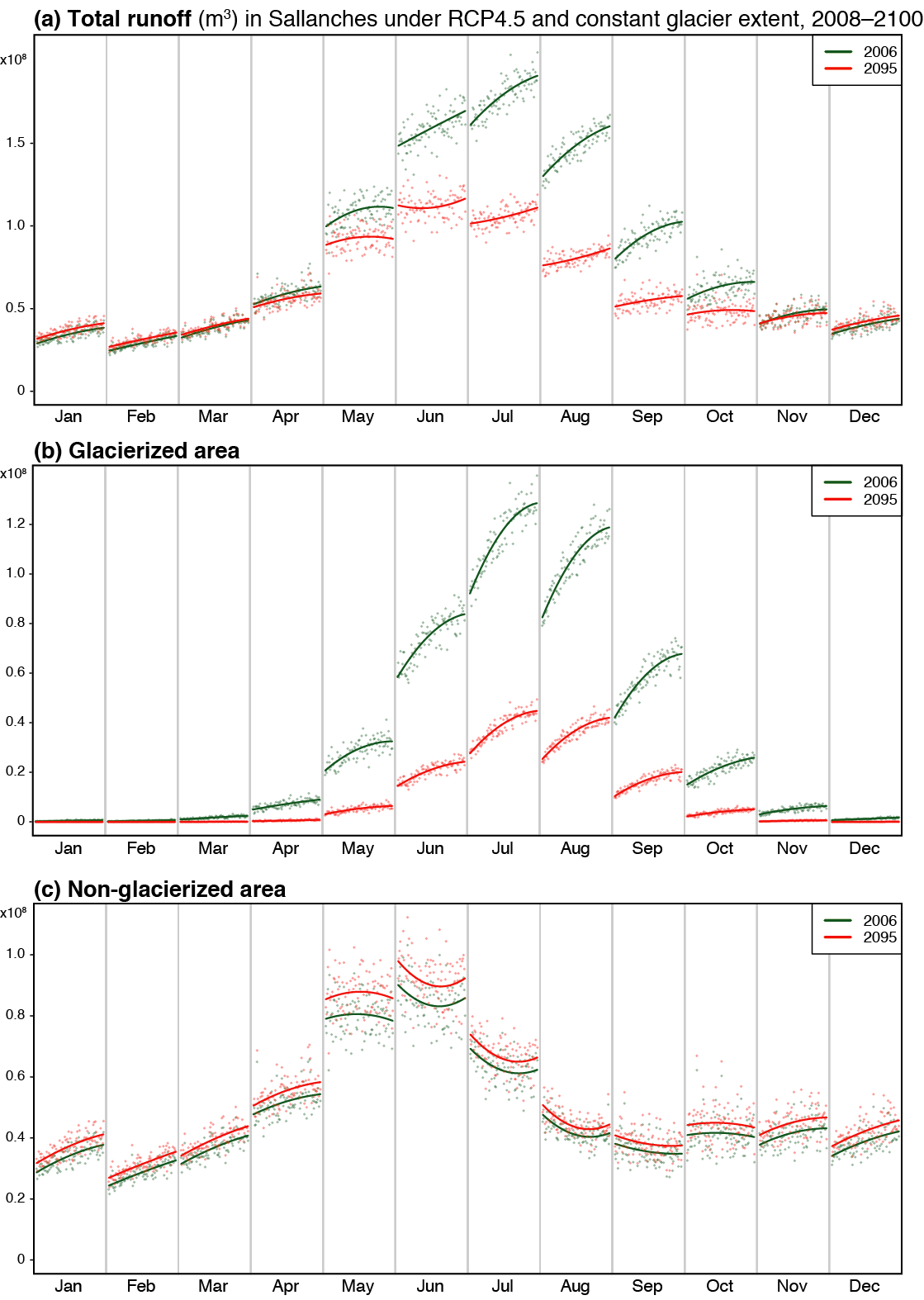


**Supplementary Figure S10 |** Monthly simulated runoff under constant glacier extension and RCP4.5 climate forcing, period 2008-2100. **(a)** Total runoff (m^3^). **(b)** Runoff from the ice-covered part (m^3^). **(c)** Runoff from the ice-free part (m^3^). Every month’s section is a 2008-2100-time series of discharges. Green colours: RCP4.5 simulations with 2006 glacier extension. Red colours: the same with a 2095 glacier extension.


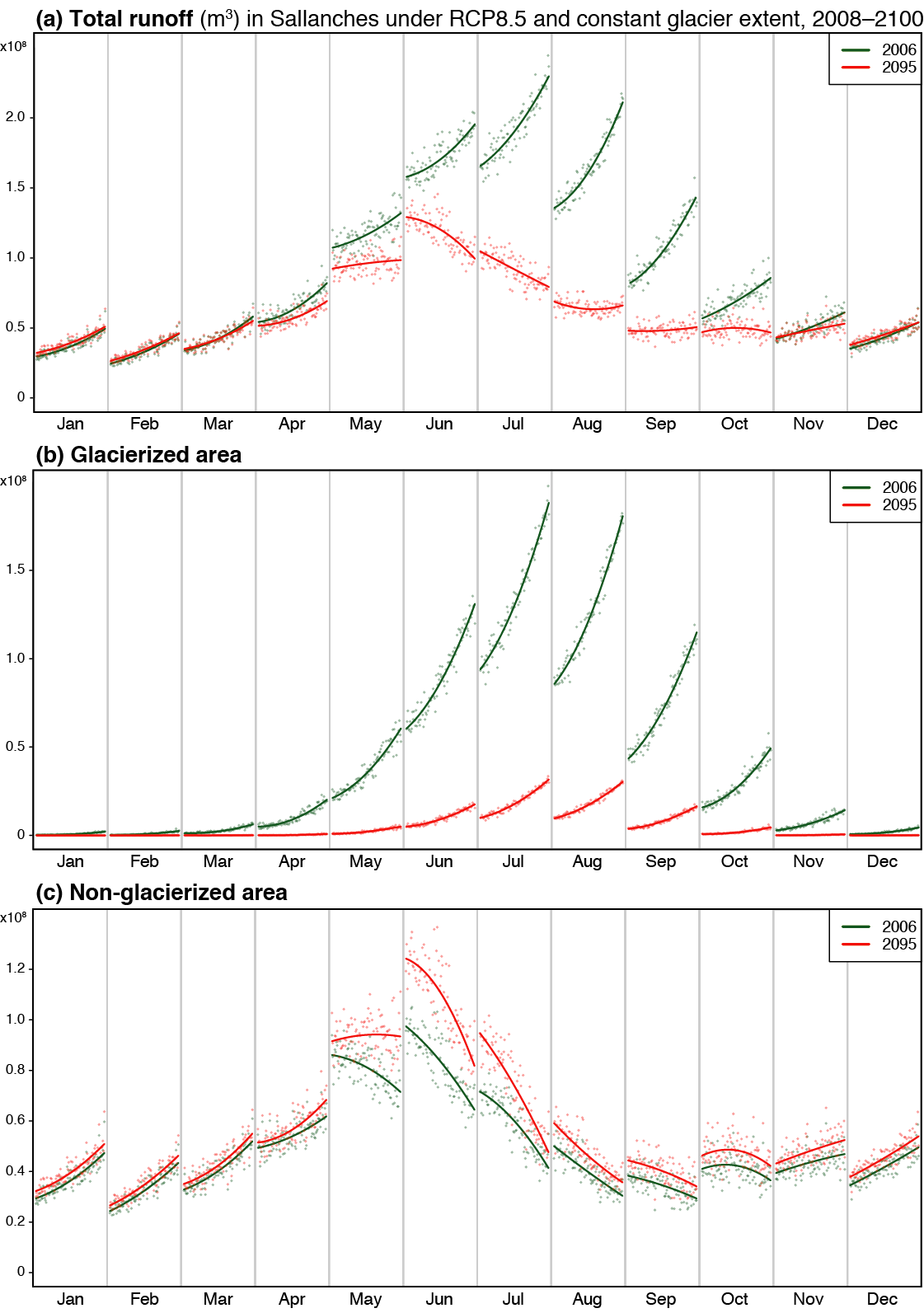


**Supplementary Figure S11 |** As Supp. Fig. S10 but under RCP8.5 climate forcing.

## 6. Disentangling climate change and glacier retreat effect


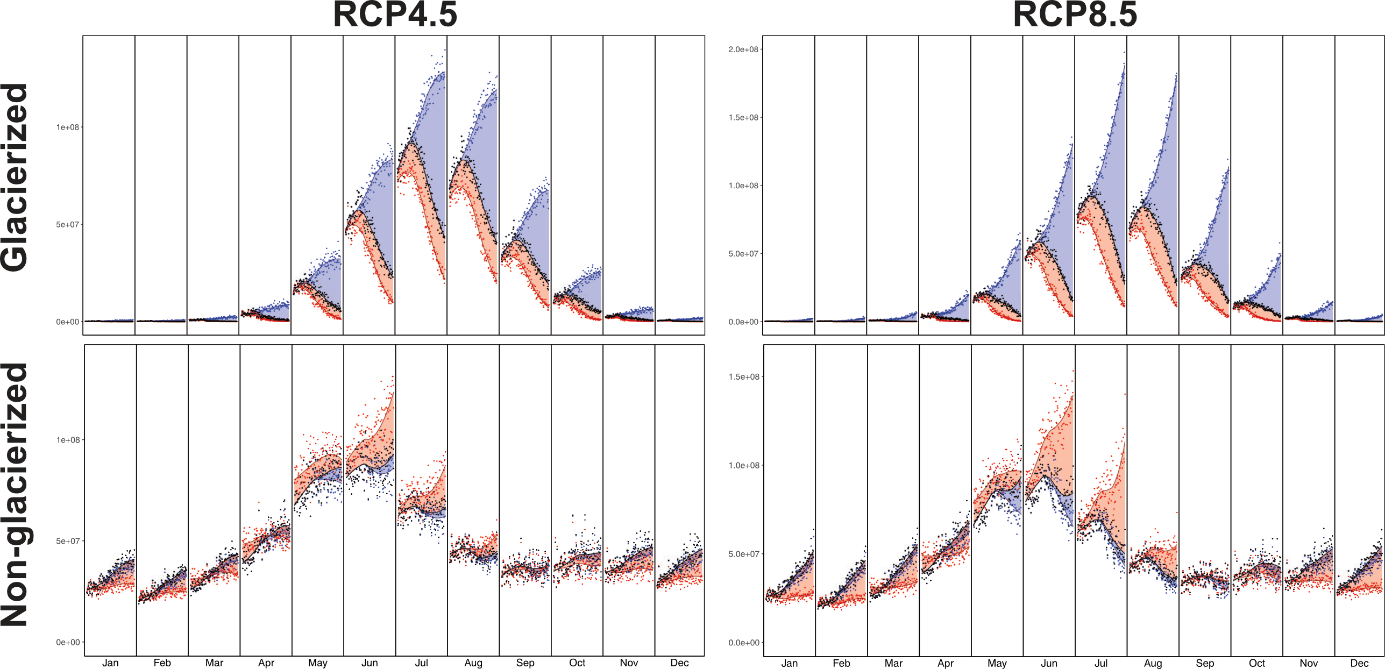


**Supplementary Figure S12 |** Monthly runoff in Sallanches (m^3^) under RCP4.5 and RCP8.5 and for glacierized and non-glacierized part of the catchment. Every month’s section is a 1967-2100-time series of discharges. Black colours: transient simulations with climate changing and glacier retreat. Each point represents the runoff for each year, the curve shows the smoothed values. Blue colours: the same for idealized simulations with climate changing and glacier constant. Red colours: the same for idealized simulations with climate constant and glacier retreat.

Relative influence of glacier retreat and climate change on total catchment runoff is presented in the main paper (**Fig. 4**). Runoff from the ice-covered part of the catchment follows the same evolution as total runoff in summer, with transient runoff mostly driven by climate change (blue curve above black curve) until 2020, and then by glacier retreat until the end of the century (red curve under black curve) (**Supp. Fig. S12**) under both forcings. By contrast, runoff coming from the non-glacierized part of the catchment computed under climate constant (red curve) is above transient runoff (black curve) in May, June, July and August under RCP4.5 and RCP8.5 (**Supp. Fig. S12**). This clearly individualizes the role of evapotranspiration: under stationary climate conditions, evapotranspiration remains stable even in summer, which leads to a higher amount of water coming from the direct runoff on the non-ice-covered part. As non-ice-covered area increases due to glacier retreat in the future, this phenomenon could explain the evolution of total runoff in June under RCP4.5 and in June and July under RCP8.5 at the end of the century (**Fig. 4**).


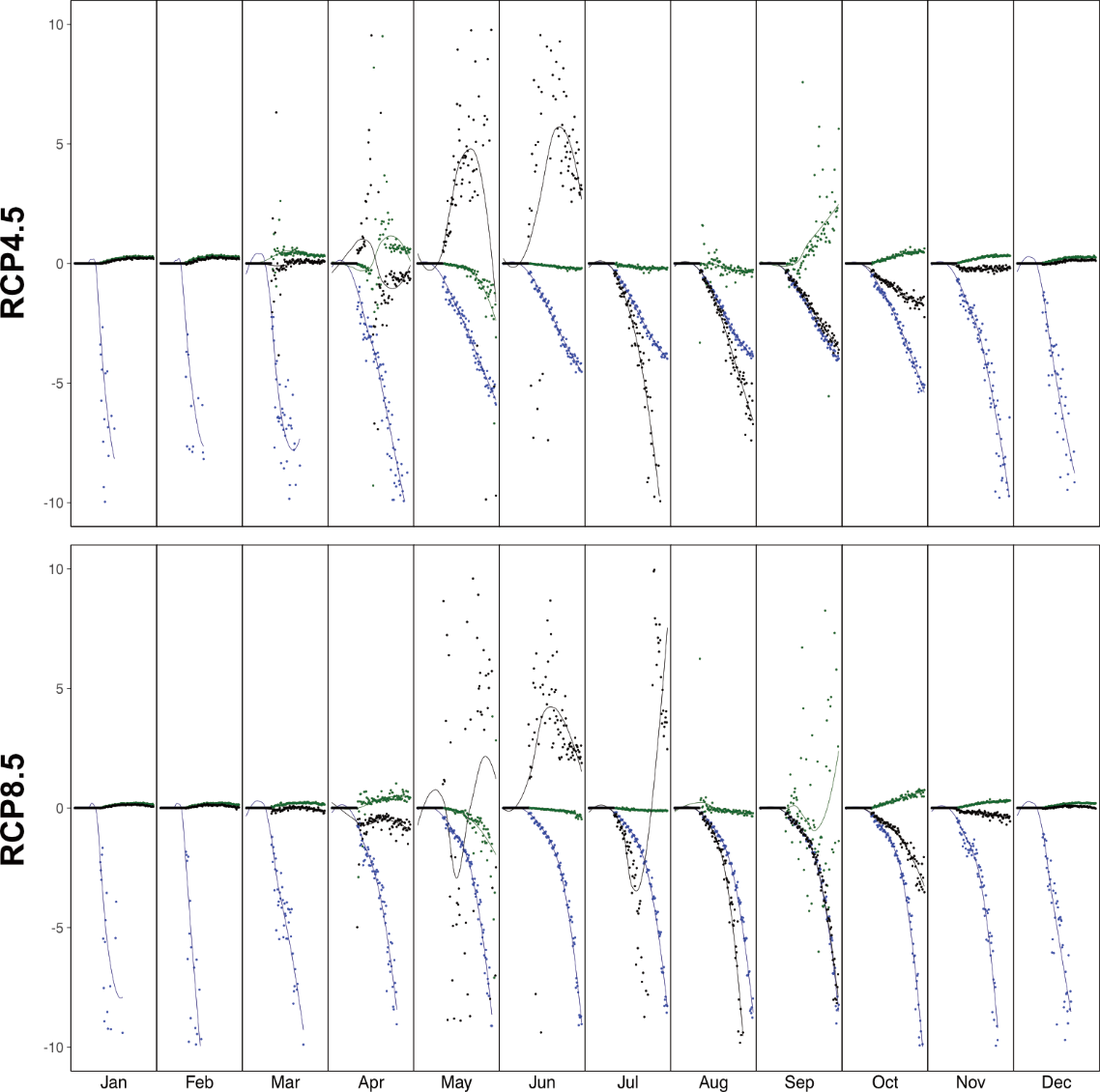


**Supplementary Figure S13 |** Monthly ratio in Sallanches (m^3^) under RCP4.5 and RCP8.5. Every month’s section is a 1967-2100-time series of discharges. Black colours: total catchment. Each point represents the ratio for each year, the curve shows the smoothed values. Blue colours: the same for glacierized part of the catchment. Red colours: the same non-glacierized part of the catchment. Ratio is the difference between runoff computed with glacier constant and transient runoff divided by the difference between runoff computed with climate constant and transient runoff.

This relative influence of glacier retreat and climate change can be estimated through the ratio between, on the one hand, the difference between runoff computed with glacier constant and transient runoff, and on the other hand, the difference between runoff computed with climate constant and transient runoff. Significant influence of glacier retreat in the future period is highlighted out through this analysis, showing that total catchment absolute value ratio for the total catchment rises in June, July, August and September under both RCPs (**Supp. Fig. S13**). Black curves increase in a positive way in June under RCP4.5 and in June and July under RCP8.5 up to 2100, supporting the larger impact of evapotranspiration on runoff evolution by the end of the century. Blue curves decrease all year long under both forcings confirming that transient runoff coming from the ice-covered part of the catchment is mostly driven by glacier retreat by 2100. On the contrary, green curves diverge only slightly from 0, pointing out the larger influence of climate change and evapotranspiration on runoff coming from the non-ice-covered part of the catchment in the future (**Supp. Fig. S13**).

In this section, new idealized simulations are produced to further disentangle the relative weight of glacier retreat and temperature increase in the long-term changes in water discharge, both driving phenomena occurring simultaneously in the real world, albeit with a different characteristic response time (Supp. Fig. S12 and S13). Temperatures used as inputs here are observations from 1968 to 2000, plus a temperature perturbation (from +0 to +5.6°C every 0.2°C). Idealized glacier extension is computed by cutting off elevation band one by one every 150m from the 2004 frontal elevations and glacier areas. In these experiments, changes in temperature and glacier area are thus defined in a completely artificial way. Mean seasonal runoff is computed over a 31-year period for each combination of temperature and glacier extension. Since precipitation amounts are statistically stationary in the current period, and show only weak changes even by the end of the century under RCP8.5, we consider this forcing as constant in these experiments, and do not assess sensitivity of simulated runoff to idealized precipitation changes. Hence, we use the observations from 1965 to 2018 as forcing conditions. Results are shown in **Supp. Fig. S14**.

CMIP5 simulations runoff under RCP4.5 and RCP8.5 from 2006 to 2100 are also displayed in **Supp. Fig. S14**, in order to show the trajectory used by these realistic changes in the two-dimension space determined by the idealized prescriptions of warming and glacier size. Yet, idealized climate forcings are not fully comparable to CMIP5 climate forcing because of the use of a simple artificial perturbation of air temperature. Thus, runoff computed using CMIP5 simulations (green and red curves) differ from runoff computed under idealized scenarios (blue colours). Total runoff simulated using RCP forcings are at most 30% lower than idealized runoff in summer (blue colours), and at most 30% higher in winter. Runoff from the ice-covered part of the catchment are at most 50% lower under RCP4.5 and RCP8.5 than under idealized scenarios. Differences between idealized scenarios and CMIP5 simulations are higher in winter due to very low runoff during this season. Runoff from the not-ice-covered part of the catchment are at most 30% higher than idealized runoff. Correlation between idealized runoff and CMIP5 simulated runoff is thus not perfect, but **Supp. Fig. S14** shows the trend of runoff evolution under temperature and frontal elevation effects.


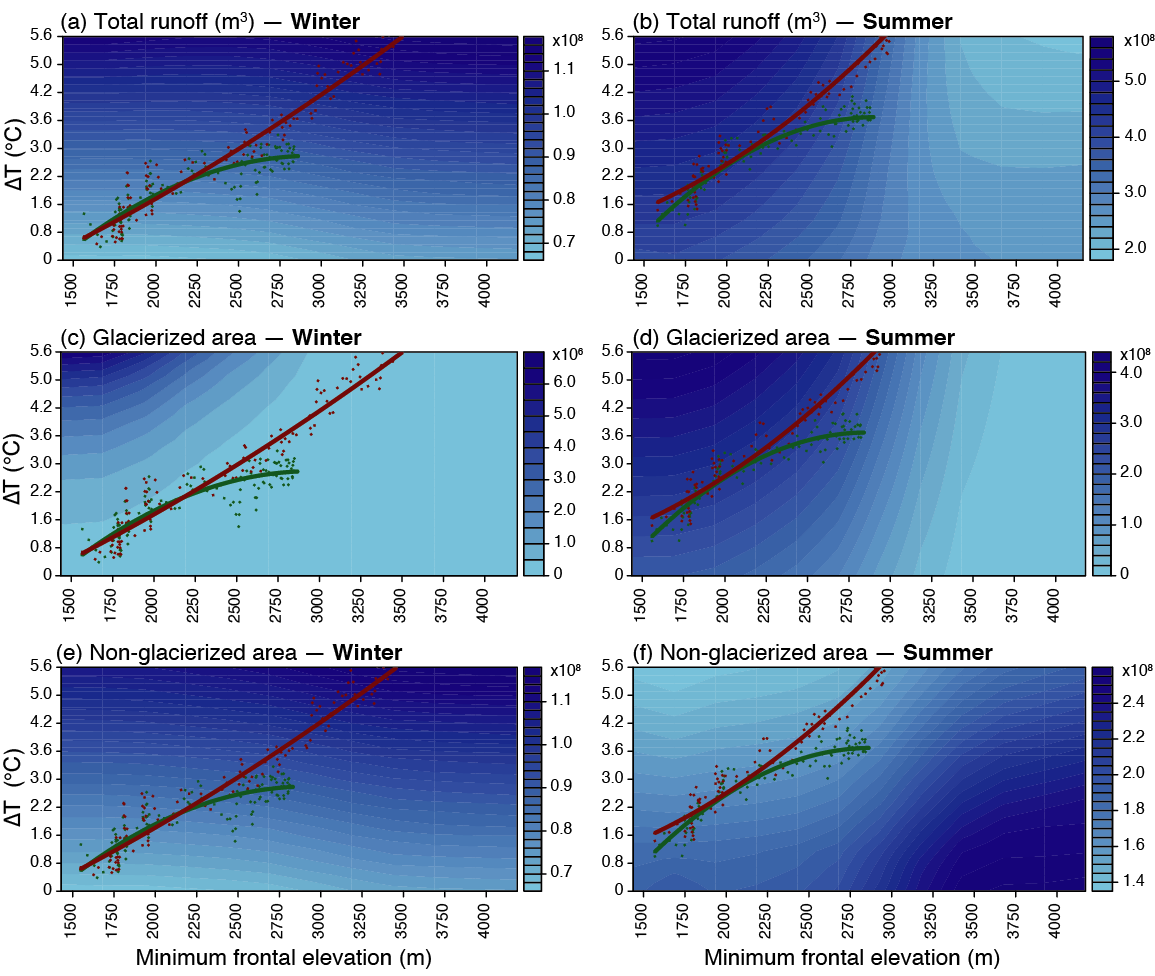


**Supplementary Figure S14 |** Mean simulated runoff over 31 years under different idealized frontal elevation (x-axis) and temperature forcing (y-axis). **(a)(b)** Total runoff (m^3^). **(c)(d)** Runoff from the ice-covered part (m^3^). **(e)(f)** Runoff from the ice-free part (m^3^). Green curve: RCP4.5 corresponding simulations. Red curve: RCP8.5 corresponding simulations.

Runoff is highly sensitive to temperature and glacier retreat (**Supp. Fig. S14**), confirming the competitive effects between these two inter-dependent factors, as discussed in the main paper and above. Prescribing a gradual temperature increase and a gradual retreat of the glacier independently from each other allows us to separate the specific influences of these two factors. In winter for example, water discharge increases due to the marked warming that causes runoff over the ice-free part of the watershed. Winter runoff would rise by 70% under the only influence of warming (**Supp. Fig. 14a**). No discharge originates from the ice-covered area, which is also more elevated and thus colder. In contrast, in summer, runoff changes under the combined influence of both temperature and glacier evolutions. Runoff increases by 40% with the only effect of warming, and decreases by 50% with the only effect of the decrease of frontal elevation (**Supp. Fig. 14b**). The contribution of the ice-covered area is predominant in June, July and August. Runoff from the ice-covered area evolve the same way in winter and summer, increasing with temperature and decreasing with frontal elevation (**Supp. Fig. 14c, d**). The highest the frontal elevation, the smallest the ice-covered part: thus, less water comes from this part of the catchment when glaciers retreat. Similarly, the highest the temperature, the more intense the ice melting, acting thus to enhance the contribution from the glacierized part of the watershed. Runoff from the ice-free part is only sensitive to temperature in winter, while it also depends on frontal elevation in summer (**Supp. Fig. 14e, f**). This illustrates the inter-dependency between glaciers, climate and runoff, and the need to perform such experiments to separate their respective influences.

Overlaying runoff simulated with CMIP5 climate inputs allows us to highlight the combined effects of temperature and ice melt on runoff evolution by 2100. It is also a graphical way to replace the transient simulations forced by two contrasted RCPs in a broader picture of possible environmental evolutions in and around the Mont-Blanc Massif. Runoff increases in winter and decreases in summer together with ongoing warming and increase in frontal elevation, systematically with larger magnitudes in the changes under RCP8.5 than RCP4.5.

# Supplementary References

1. Leroy, M. La mesure au sol de la température et des précipitations. Météorologie 8, 52 (2002).
2. Thornthwaite, C. W. An Approach toward a Rational Classification of Climate. Geographical Review 38, 55–94 (1948).
3. Zekollari, H., Huss, M. & Farinotti, D. Modelling the future evolution of glaciers in the European Alps under the EURO-CORDEX RCM ensemble. *The Cryosphere* **13**, 1125–1146 (2019).
4. Brunner, M. I. *et al.* Present and future water scarcity in Switzerland: Potential for alleviation through reservoirs and lakes. *Science of the Total Environment* **666**, 1033–1047 (2019).
5. Brunner, M. I., Farinotti, D., Zekollari, H., Huss, M. & Zappa, M. Future shifts in extreme flow regimes in Alpine regions. Hydrology and Earth System Sciences 23, 4471–4489 (2019).
6. Marzeion, B. et al. Partitioning the Uncertainty of Ensemble Projections of Global Glacier Mass Change. Earth’s Future n/a, (2020).
7. Intergovernmental Panel on Climate Change. *Climate Change 2013: The Physical Science Basis. Contribution of Working Group I to the Fifth Assessment Report of the Intergovernmental Panel on Climate Change.* 1585 (2013).
8. Colette, A., Vautard, R. & Vrac, M. Regional climate downscaling with prior statistical correction of the global climate forcing. *Geophysical Research Letters* **39**, (2012).
9. Michelangeli, P. A., Vrac, M. & Loukos, H. Probabilistic downscaling approaches: Application to wind cumulative distribution functions. *Goephysical Research Letters* **36**, (2009).
10. Schaefli, B., Hingray, B., Niggli, M. & Musy, A. A conceptual glacio-hydrological model for high mountainous catchments. *Hydrology and Earth System Science* **9**, 95–109 (2005).
11. Her, Y., Yoo, S.H., Cho, J., Hwang, S., Jeong, J. & Seong, C. Uncertainty in hydrological analysis of climate change: multi-parameter vs. multi-GCM ensemble predictions. Scientific Reports **9**, 4974 (2019).
12. Baker, D., Escher-Vetter, H., Moser, H. & Oerter, H. A glacier discharge model based on results from field studies of Energy balance, water storage and flow. *Hydrological Aspects of Alpine and High Mountains Areas* **138**, 10 (1982).
13. Consuegra, D. & Vez, E. *AMIE – Analyse et Modélisation Intégrées du cheminement des Eaux en zones habitées, modélisation hydrologique, Application au bassin versant de la Haute Broye*. (1996).
14. RGI Consortium. Randolph Glacier Inventory – A Dataset of Global Glacier Outlines: Version 6.0: Technical Report, Global Land Ice Measurements from Space, Colorado, USA. *Digital Media* (2017)
15. World Meteorological Organization. Intercomparison of models of snowmelt runoff. 482 https://library.wmo.int/doc_num.php?explnum_id=1686 (1986).
16. Rango, A. & Martinec, J. Revisiting the degree-day method for snowmelt computations. *Journal of the American Water Ressources Association* **31**, 657–669 (1995).
17. Singh, P., Kumar, N. & Arora, M. Degree–day factors for snow and ice for Dokriani Glacier, Garhwal Himalayas. *Journal of Hydrology* **235**, 1–11 (2000).
18. Hock, R. Temperature index melt modelling in mountain areas. *Journal of Hydrology* **282**, 104–115 (2003).
19. Klok, E. J., Jasper, K., Roelofsma, K. P., Gurtz, J. & Badoux, A. Distributed hydrological modelling of a heavily glaciated Alpine river basin. *Hydrological Sciences Journal* **46**, 553–570 (2001).
20. Consuegra, D., Niggli, M. & Musy, A. Concepts méthodologiques pour le calcul des crues. Application au bassin versant du Rhône. *Wasser Energie Luft* **90**, 223–231 (1998).
21. Guex, D., Guex, F., Pugin, S., Hingray, B. & Musy, A. *Regionalisation of hydrological processes in view of improving model transposability. WP2 Final Report of the Pan-European FRHYMAP Project (Flood Risk scenarios and Hydrological MAPping), N° Contract CE : 3/NL/1/164/99 15 183 01*. (2002).
22. Braithwaite, R. J. & Olesen, O. B. Calculation of Glacier Ablation from Air Temperature, West Greenland. *Glacier Fluctuation and Climatic Change* **6**, 219–233 (1989).
23. Swift, D. A., Nienow, P. W., Hoey, T. B. & Mair, D. W. F. Seasonal evolution of runoff from Haut Glacier d’Arolla, Switzerland and implications for glacial geomorphic processes. *Journal of Hydrology* **309**, 133–148 (2005).
24. Pohl, B. *et al.* Huge decrease of frost frequency in the Mont-Blanc massif under climate change. *Scientific Reports* **9**, (2019).
